# Transitioning to Civilian Life: How Are Life Meaning, Purpose and Identity Related to Wellbeing for Veterans? A Scoping Review

**DOI:** 10.3390/bs16081386

**Published:** 2026-08-12

**Authors:** Cassandra Walters, Lauren Miller-Lewis, Amy Johnson

**Affiliations:** 1 College of Psychology, Central Queensland University, Rockhampton 4701, Australia; l.miller-lewis@cqu.edu.au; 2 Cluster for Resilience and Wellbeing, Appleton Institute, Central Queensland University, Rockhampton 4701, Australia; a.johnson2@cqu.edu.au; 3 College of Arts, Central Queensland University, Rockhampton 4701, Australia

**Keywords:** veteran, military-to-civilian transition, identity, purpose, meaning in life, wellbeing

## Abstract

Veterans transitioning out of full-time military service to civilian life face unique challenges which may adversely shape their wellbeing, including complex changes in a veteran’s sense of identity and experiences of meaning and purpose in life. There is an evidence-synthesis gap regarding how transition affects veterans’ sense of identity, life meaning, purpose and overall wellbeing. A pre-registered scoping review of empirical studies was conducted using the Joanna Briggs Institute Scoping Review methodology. Overall, fifteen studies met the inclusion criteria. Key themes were identified in the evidence relating to the role of identity and life meaning and purpose in veteran wellbeing. The review found that veterans frequently report identity disruption and loss of meaning or purpose in life after transition, and these experiences were associated with lower wellbeing. Strongly held military identity, particularly viewing the military as family, was also associated with poorer wellbeing and adjustment in several studies. The review highlights the complex nature of transition and how it influences a veteran’s sense of identity, life meaning and purpose, and wellbeing. Military service can provide a strong sense of meaning and purpose to life, but transition may require veterans to renegotiate identity and develop new sources of life meaning and purpose. Further research is required to deepen understanding of veterans’ wellbeing needs, thereby informing the development of targeted supports that facilitate a successful transition to civilian life.

## 1. Introduction

When military personnel leave service, they face more than just a job change, given military service can provide personnel with a clear purpose and meaning. Service impacts extend beyond military transition—there are often greater risks to a veteran’s mental and physical health and wellbeing following their transition from full-time service (Bryant et al., 2019; Department of Defence, 2017; Department of Veterans’ Affairs & Department of Defence, 2018; Flack & Kite, 2021; Lawrence-Wood et al., 2019; Royal Commission into Defence and Veteran Suicide, 2024; Van Hooff et al., 2018). It is crucial to understand the evidence-based psychosocial factors that may influence veterans’ wellbeing. The transition period poses significant challenges due to the complete shift in culture that a veteran goes through when leaving the military, which is often referred to as a ‘reverse culture shock’ (Pedlar et al., 2019). Military service shapes a person’s entire worldview, their perceptions of self and others (Keeling, 2024). It is described as a ‘total’ (Shepherd et al., 2021) or ‘greedy’ (Segal, 2025) institution, shaped by a unique culture which is based on beliefs, behaviours, practices, norms, tradition and community (Heward et al., 2024; Sachdev & Dixit, 2024). This psychosocial journey that veterans go through whilst serving permeates their beliefs, emotions and behaviours, and this deep permeation generates internal conflict when entering back into the civilian world (Heward et al., 2024). The impact of military transition on mental illness is well-recognised (Royal Commission into Defence and Veteran Suicide, 2024; U.S. Government Accountability Office [GAO], 2025). For example, the concern about veteran mental illness contributed to the establishment of an Australian Royal Commission into Defence and Veteran Suicide (RCDVS) in 2021. This inquiry is but one policy example of growing multinational concern about transition and veteran wellbeing.

Within the discipline of positive psychology, mental illness and wellbeing are considered conceptually distinct (Keyes, 2002). The concept of wellbeing is often misunderstood as simply an individual having a lack of psychopathology (i.e., PTSD, anxiety, depression) in their life (Park et al., 2021), and that absence is sufficient for individuals to feel fulfilled and flourish. But wellbeing is far more than just the absence of mental illness. Theorists such as Keyes (2002) state that mental illness and wellbeing sit on separate continuums that combine to generate a picture of ‘complete mental health’. This dual-continua recognises that a person with a diagnosed mental illness can still experience positive wellbeing, and a person without mental illness can languish with low wellbeing (Snyder & Lopez, 2002). Wellbeing is defined by the World Health Organization (WHO) “as a positive state experienced by individuals and societies. Similar to health, it is a resource for daily life and is determined by social, economic, and environmental conditions. Wellbeing encompasses quality of life, as well as the ability of people and societies to contribute to the world in accordance with a sense of meaning and purpose” (World Health Organization, 2021, p. 10). Having a strong sense of identity lays the foundation for meaning in life (Davies et al., 2023; Jarden & Roache, 2023).

Emerging bodies of research suggest the challenges faced during transition influence veteran wellbeing (Flack & Kite, 2021), not just mental illness. For example, the RCDVS acknowledged that transition is a time of great change and is frequently associated with loss of purpose and identity (Royal Commission into Defence and Veteran Suicide, 2024). Indeed, a significant contributing component of an individual’s overall wellbeing is their sense of identity and experiences of meaning and purpose in life. A person’s identity is formed as they experience and engage in their cultural context (Kerr et al., 2024). Someone’s personal identity refers to how an individual defines themselves, the ‘I’ and ‘me’ as a unique construct that differs from other members in a group (Barnett et al., 2022). The shift from ‘we to me’ (Kerr et al., 2024) is seen as a pivotal part of a veteran’s transition out of the military, as the inculcation into the military identity is vastly different to civilian occupations. The identity which forms during a military career has even been suggested to be so all-consuming that it is akin to ethnicity, enmeshing so much with an individual’s sense of self (Dolan et al., 2022). It is widely accepted that the military identity is a social identity which is formed as the civilian identity becomes less salient through military acculturation (Tkachuck et al., 2022) and is further integrated into the self-concept (Dolan et al., 2022; Flack & Kite, 2021; Moeck et al., 2022). Identity, meaning in life and purpose are conceptually related but distinct. Identity helps locate a person within valued roles, groups and narratives of self. Meaning in life is defined as our understanding of our lives as (a) having value and significance, (b) making coherent sense, and (c) having direction or purpose, with purpose being a future-oriented facet of meaning in life that provides direction for future action (Martela & Steger, 2016). During military-to-civilian transition, these internal representations might be disrupted because leaving service may involve loss of group belonging, role clarity and service-oriented meaning and purpose in life (Davies et al., 2023; Dolan et al., 2022; Kerr et al., 2024; Martela & Steger, 2016). Examining these constructs together therefore enables a more integrated understanding of wellbeing than considering either identity, life meaning or purpose in isolation.

### Review Objectives

There is a need to better understand the contemporary evidence base regarding how identity, meaning and purpose in life relate to wellbeing during military-to-civilian transition. Existing reviews have examined similar but adjacent topics, including focusing on mental illness outcomes rather than wellbeing (Shields et al., 2016), military/veteran identity salience post-discharge rather than wellbeing (Dolan et al., 2022), cultural transition experiences in qualitative studies (Sachdev & Dixit, 2024), and veteran women’s wellbeing experiences in qualitative studies (Jones & Hanley, 2017). These adjacent reviews have made important contributions to knowledge, but there remains a lack of comprehensive exploration mapping and synthesising the qualitative and quantitative evidence base regarding identity, life meaning and purpose as potential mechanisms through which transition may shape veteran wellbeing. This scoping review aims to address that gap, shifting attention beyond a deficit-focused mental illness narrative onto wellbeing outcomes that enable a more preventative approach, and generate insights relevant across comparable transition systems rather than only within one national context (e.g., Royal Commission into Defence and Veteran Suicide, 2024).

The objective of this scoping review is to answer the following research question: for veterans transitioning out of military service, how does their sense of identity, life meaning and purpose relate to their wellbeing? Specifically, it sought to scope the existing literature to evaluate: (a) What evidence exists about how identity, meaning and purpose in life are associated with wellbeing in veterans? (b) What types of empirical studies have been conducted? (c) What countries are represented in the research? (d) Has the term veteran been defined in the research? (e) Was time since discharge considered? (f) How have identity, purpose/meaning in life, wellbeing and transition been defined in the research? (g) Was the role of biological sex or gender roles/norms considered?

## 2. Method

A scoping review was chosen due to its design enabling the mapping of available evidence against a series of broad questions, including scoping study designs and definitions alongside study findings (Pollock et al., 2024). The scoping review was conducted in accordance with the Joanna Briggs Institute (JBI) scoping review methodology (Peters et al., 2020) and the Preferred Reporting Items for Systematic Reviews and Meta-Analysis Guidelines for scoping reviews, PRISMA-ScR (Page et al., 2021; Tricco et al., 2018). The review protocol was pre-registered with OSF Registries: https://osf.io/3es47, accessed on 19 May 2026.

### 2.1. Search Strategy

A research librarian optimised the search strategy in the following databases: Embase and Medline (Elsevier); PsycINFO (Ovid); CINAHL (EBSCOhost); and grey literature databases including Scopus, Google Scholar, ProQuest Dissertation and Theses Global and Open Access Theses and Dissertations. The Grey Literature searches used combinations of the main keywords (veteran, transition, wellbeing, identity, purpose). Searches were conducted in September 2024. The literature database search strategy, including search strings, can be found in Supplementary Table S1. No database limiters for publication language or year were used.

### 2.2. Inclusion and Exclusion Criteria

Articles in this scoping review needed to meet the inclusion criteria outlined in Table 1. Eligible studies examined veteran identity and/or purpose/meaning in life in relation to wellbeing, or closely related indicators such as life satisfaction and quality of life (QoL). Given the ongoing debates about how best to conceptualise wellbeing (Iasiello et al., 2024; Jarden & Roache, 2023), a broad approach was used when selecting search terms to ensure no omissions of potentially relevant studies examining positively oriented wellbeing-related outcomes. Studies with a deficit-oriented focus solely on psychopathology or mental distress without attention to broader positive psychosocial adjustment were excluded. To guide the review, we adopted Moeck et al.’s (2022) definition of a veteran as a former member of a nation’s defence force, being active or reserve force, regardless of service length. No restrictions were placed on service type or length of service, or on empirical study type. Non-empirical articles, articles published prior to 2000, and articles published in a language other than English were excluded at the screening stage.

### 2.3. Screening and Study Selection Process

All authors were involved in screening using the COVIDENCE platform (Melbourne, Australia) to ensure consistent application of study eligibility criteria. Each source was independently assessed by at least two raters, with a third adjudicator if required. Inter-rater agreement at the abstract stage was 92.5%, and 76.5% at the full-text stage. All discrepancies were resolved through adjudication and discussion. Hand searching of reference lists contained in the included studies was undertaken in conjunction with the structured search to ensure all relevant research was included. Handsearching produced duplicate records and no additional eligible studies were found. The screening and selection process was recorded on the PRISMA flowchart (Page et al., 2021; Tricco et al., 2018).

### 2.4. Data Extraction and Data Synthesis

The data extracted from eligible studies were recorded in Supplementary Table S2. Data extraction items included author(s), publication year and title, study aims/research question(s), study setting and sample size, sample characteristics (e.g., reported gender or sex, age), study design and method, key constructs, exposure and outcome variables (and how they were examined), main study findings including quantitative effect sizes (if reported-if not reported by the included studies, they were not inferred or calculated for this scoping review), and qualitative themes identified by studies and how they mapped to identity, purpose/meaning, and wellbeing. Additionally, we documented article definitions used for key concepts to enable a comparative analysis across studies. As per scoping review methodology, critical appraisal tools were not formally used to assess the methodological quality of the included studies (Peters et al., 2020; Pollock et al., 2024). Nonetheless, we recorded information on study strengths, limitations and future directions. These methodological strengths and limitations were considered when interpreting findings and developing the narrative synthesis, with greater weight given to evidence with lower risk of bias. Finally, the study results were charted and coded to identify common patterns and themes. First, study findings were charted by construct (identity, life meaning/purpose, and wellbeing/adjustment). Second, the first author inductively coded recurring patterns from the reported findings and compared them across study designs. Third, codes were grouped into higher-order identity and life meaning/purpose themes. The second author checked the coding and theme structure, and variations in interpretation were resolved through discussion. The resulting thematic codes enabled a narrative synthesis of the evidence regarding how identity and life meaning/purpose are related to veteran wellbeing (Arksey & O’Malley, 2005; Peters et al., 2015, 2020; Rodgers et al., 2009).

## 3. Results

### 3.1. Study Selection

**Figure 1:**
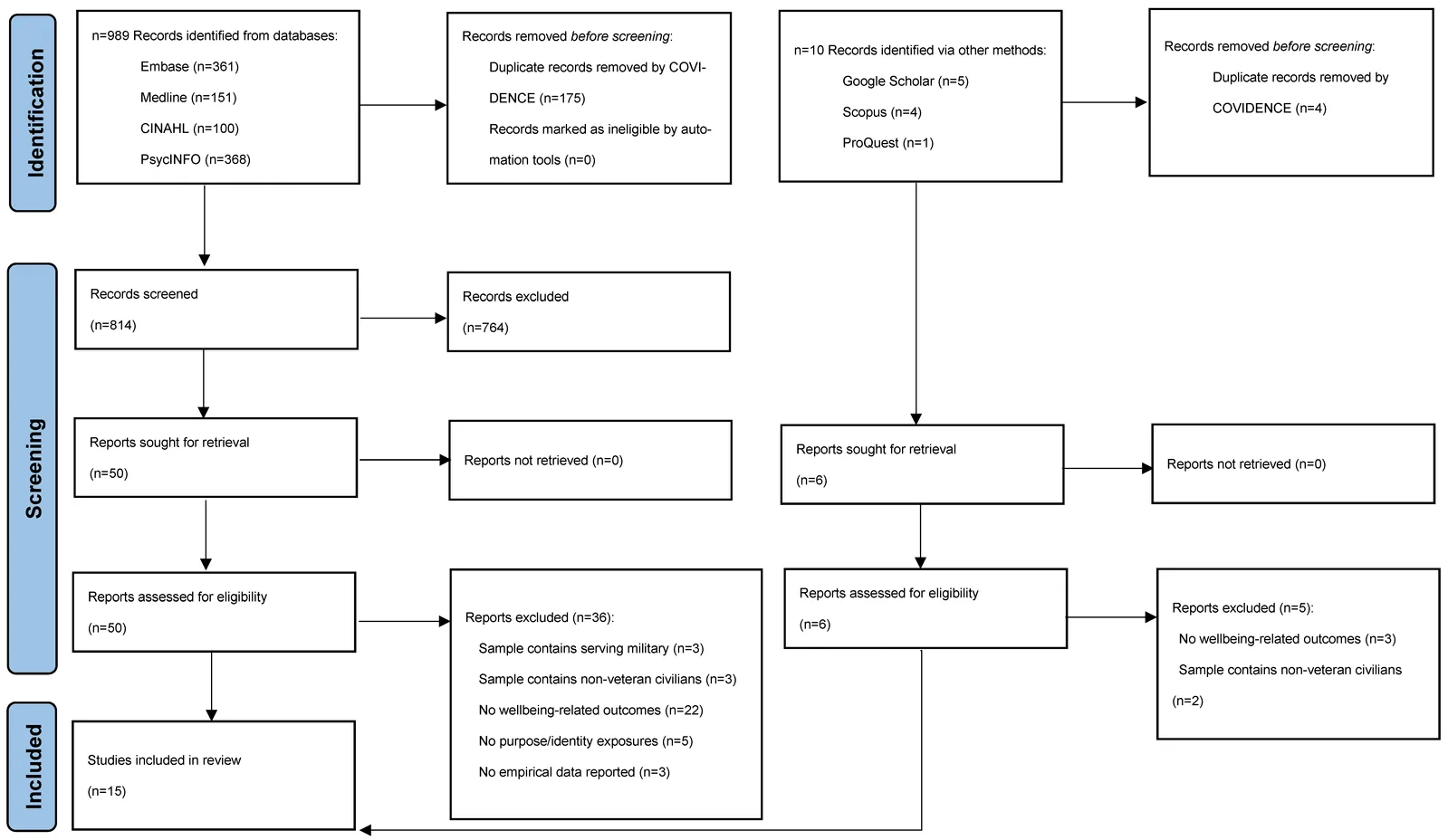
PRISMA (Preferred Reporting Items for Systematic Reviews and Meta-Analyses) flow diagram of the study selection process.

Database searches returned 989 articles for screening. An additional 10 potential articles were identified through other sources. A total of 15 empirical articles were included for the review. Figure 1 presents the search and selection process according to PRISMA guidelines.

Data was systematically extracted from the 15 studies meeting eligibility criteria. Key features relevant to the scoping review research questions are summarised below. A concise overview of study characteristics is also provided in Table 2. Supplementary Table S2 provides full study-by-study detailed extraction information for each included study, including country, sample characteristics, study design, constructs measured, available transition timing information, key findings, and study methodological strengths and limitations. Below, study characteristics and conceptual definitions used in the studies are reported (Section 3.2) followed by the narrative synthesis of study findings (Section 3.3).

### 3.2. Characteristics of Included Studies

#### 3.2.1. What Countries Are Represented in the Research?

All 15 eligible studies were published between 2001 and 2024 and originated from English-speaking countries, predominantly the United States of America (*n* = 7) (Burkhart & Hogan, 2015; Gnall et al., 2023; Keeling, 2024; Lancaster et al., 2018; Mitchell et al., 2020; Park et al., 2021; Stevenson, 2020). Three studies were conducted in Australia (Barnett et al., 2022; Carra et al., 2023; Flack & Kite, 2021), two originated from Canada (Lee et al., 2020; Waldhauser et al., 2021), and three were from the United Kingdom (Brewster et al., 2021; Reed, 2001 Wakefield et al., 2024).

#### 3.2.2. Has the Term ‘Veteran’ Been Defined in the Research?

Only 2 of the 15 studies offered a clear definition of the term ‘veteran’. Both articles that defined what it means to be a veteran acknowledged that it was a person who had previously served in the military and was released under honourable conditions (Brewster et al., 2021; Waldhauser et al., 2021). These studies were from the United Kingdom and Canada, respectively.

#### 3.2.3. Was Time Since Discharge Considered?

Eight studies (Barnett et al., 2022; Carra et al., 2023; Flack & Kite, 2021; Gnall et al., 2023; Keeling, 2024; Lee et al., 2020; Park et al., 2021; Reed, 2001) recorded time since discharge. The time since discharge varied between one year and 12 years across these studies. One study spoke about time since deployment (Mitchell et al., 2020) being six years, but it was not clear if this meant time since deployment on operations. The other six studies (Brewster et al., 2021; Burkhart & Hogan, 2015; Lancaster et al., 2018; Stevenson, 2020; Wakefield et al., 2024; Waldhauser et al., 2021) did not mention time since discharge. Because the available studies varied in transition timing, the evidence should be understood as spanning acute transition, longer-term post-discharge adjustment and broader veteran wellbeing. This means study findings can only be considered within the transition timeframes captured across the included studies, and cannot be interpreted as applying uniformly across all stages of civilian reintegration, particularly where time since discharge was not reported.

#### 3.2.4. What Types of Empirical Studies Have Been Conducted?

The included studies were heterogeneous in study design and methodological quality, with sample sizes ranging from a *n* = 1 case study (Stevenson, 2020) to a representative sample of *n* = 1414 (Lee et al., 2020). Six of the studies were quantitative (Flack & Kite, 2021; Gnall et al., 2023; Lancaster et al., 2018; Lee et al., 2020; Park et al., 2021; Reed, 2001), six were qualitative (Barnett et al., 2022; Brewster et al., 2021; Burkhart & Hogan, 2015; Carra et al., 2023; Keeling, 2024; Waldhauser et al., 2021), two adopted a mixed-methods approach (Mitchell et al., 2020; Wakefield et al., 2024), and one was a case study (Stevenson, 2020). Only three studies contained a longitudinal component to track veteran outcomes over time (Gnall et al., 2023; Mitchell et al., 2020; Park et al., 2021), and thus there is limited evidence to support causal inferences. Only two studies reported using a representative sample (Lancaster et al., 2018; Lee et al., 2020). The most common data-collection methodology used in the studies was semi-structured or narrative interviews, with eight studies (Barnett et al., 2022; Brewster et al., 2021; Burkhart & Hogan, 2015; Carra et al., 2023; Gnall et al., 2023; Keeling, 2024; Park et al., 2021; Waldhauser et al., 2021) adopting this approach. Four quantitative studies (Flack & Kite, 2021; Lancaster et al., 2018; Lee et al., 2020; Reed, 2001) used surveys to capture their data, one study (Wakefield et al., 2024) used a combination of a survey and semi-structured interviews, one (Barnett et al., 2022) used a combination of interviews and a mapping task, one (Park et al., 2021) used a writing task and a survey, and one (Stevenson, 2020) used psychotherapy sessions to capture case study data. Across studies containing a qualitative component, the reported themes clustered most strongly around wellbeing and identity. Qualitative themes contained conceptual variability in the use of terms related to meaning and purpose in life.

#### 3.2.5. How Has Identity, Purpose/Meaning in Life, Wellbeing and Transition Been Defined in the Research?

The studies varied significantly in their attention to explicitly defining wellbeing, identity, life meaning/purpose, or transition, as can be seen in Supplementary Table S3. Transition was defined in only one study, where it was described as simply the move from one way of life (service-person) to another (veteran) (Wakefield et al., 2024). Six of the articles defined identity (Barnett et al., 2022; Brewster et al., 2021; Flack & Kite, 2021; Lancaster et al., 2018; Mitchell et al., 2020; Waldhauser et al., 2021). None of the articles explicitly defined life purpose, although one defined meaning in life (Carra et al., 2023). Only two articles explicitly defined wellbeing (Brewster et al., 2021; Park et al., 2021). The way in which identity was defined varied between identity in terms of ‘self-perception’ and identity in a ‘social’ context. The studies that defined wellbeing were aligned in their conceptualisation of wellbeing as more than the absence of a mental illness diagnosis, that it is rooted in social supports, meaning in life and an understanding of life in its social context (Brewster et al., 2021; Park et al., 2021). This conceptual variability also helps explain why some included studies operationalised wellbeing through broader positive-adjustment indicators such as quality-of-life rather than an explicit wellbeing scale.

The most common measurement tools used within the quantitative studies for assessing wellbeing were the World Health Organization Quality of Life BREF (The WHOQOL Group, 1998), the Satisfaction With Life Scale (Diener et al., 1985), and the Mental Health Quality of Life Short Form (Ware et al., 1996). Identity was typically measured using the Warrior Identity Scale (Lancaster et al., 2018) or a one item 5-point Likert scale measure, and life meaning/purpose was most-frequently measured using the Meaning in Life Questionnaire (Steger et al., 2006). No quantitative studies used a specific measure of life purpose.

#### 3.2.6. Was the Role of Gender or Biological Sex Considered?

The World Health Organization distinguishes sex as referring to biological characteristics, and gender referring to socially constructed roles, norms and identities (Kaufman et al., 2023). All 15 included studies reported information in relation to participant gender or biological sex, but the studies did not consistently define or distinguish gender from sex, and several did not identify whether reported categories such as male/female referred to sex assigned at birth, gender identity, or gendered social roles. Accordingly, this review uses gender when discussing social-cultural factors (such as civilian expectations of women), and uses sex only when referring to how the included studies reported participant characteristics. Most participants recruited for the studies were reported as ‘male’, with four of the studies (Keeling, 2024; Reed, 2001; Stevenson, 2020; Waldhauser et al., 2021) only recruiting male participants (with Reed, 2001, and Stevenson, 2020 referring to male gender). There was one study focused on female gender veterans (Burkhart & Hogan, 2015). Two studies referred to ‘sex’ (Lee et al., 2020; Mitchell et al., 2020), but only one study explored how ‘sex’ may shape veteran wellbeing (Lee et al., 2020). Their results indicated that females were more likely to perceive the loss of military identity as a challenge compared to males. Lee et al. (2020) suggested this may be due to the unique aspects of military careers for females, and expectations of what it is to be a civilian female (and reassume a civilian female identity as a veteran). No other conclusions were drawn from the remaining studies regarding the difference gender or biological sex may make in how identity and life meaning/purpose relate to the veteran’s wellbeing and the different influences transition may have on wellbeing based on biological sex or gender roles or identities. There is a clear evidence gap in considering veterans who do not identify as their biological sex—no studies reported if their sample contained non-binary or Transgender veterans; Flack and Kite (2021) noted a single study participant who did not disclose gender, and Wakefield et al. (2024) reported their sample contained two veterans with gender recorded as ‘other’, with no further detail provided.

### 3.3. Synthesis of Key Findings on How Identity and Life Meaning/Purpose Are Related to Veteran Wellbeing

The synthesis of key findings addresses the review question concerning what evidence exists about how identity and life meaning/purpose are associated with wellbeing in veterans. In analysing the findings of the 15 studies, relevant patterns in relation to identity and life meaning/purpose became apparent, and thus the findings were thematically synthesised in these groupings. Six themes were identified relating to identity and three related to life meaning/purpose. An overview of the themes is provided in Table 3, and they are described in detail below. Whilst formal critical appraisal of study quality was not conducted, in developing this narrative synthesis of key findings, consideration was given to the methodological strengths and limitations of the studies (outlined in Supplementary Table S2), with greater emphasis placed on longitudinal findings tracking veteran wellbeing over time.

#### 3.3.1. Strong Military Identity—Military Is My Family

As shown in Table 3, eleven of the included studies reported findings relevant to identity. Aligning with identity theories (Dolan et al., 2022; Haslam et al., 2021), the included studies demonstrated that military identity is multidimensional and complex. Across the samples in the included studies and within the transition timeframes captured (where reported), holding military identity as highly central and/or viewing the military as ‘family’ was associated with poorer subjective wellbeing, lower life satisfaction, weaker social connectedness, and greater difficulty adjusting to civilian life post-discharge (Barnett et al., 2022; Flack & Kite, 2021; Lancaster et al., 2018). Keeling (2024) further illustrated the continued importance of military values and identity in veterans’ transition narratives. These findings suggest that the way a veteran relates to the military may shape social connectedness, which in turn may influence wellbeing. Stronger and more exclusive military identity was generally associated with greater feelings of disconnection from civilian society and self-reported poorer psychosocial adjustment. If veterans reported feeling unable to form strong connections outside the military environment, they also tended to report diminished wellbeing (Barnett et al., 2022). However, causal directions cannot be inferred due to the cross-sectional nature of the available evidence, and the influence of transition stages is unclear due to timeframe variability in the studies.

#### 3.3.2. Mode of Transition Matters for Identity

Across the sampled studies, the way military members exited the military was associated with veterans’ wellbeing within the transition timeframes captured. In four studies (Barnett et al., 2022; Keeling, 2024; Lee et al., 2020; Reed, 2001), veterans who did not voluntarily choose to discharge, or who were not psychologically prepared for transition, reported greater feelings of identity loss and, in some studies, lower life satisfaction than those who chose to leave. Taken together, these findings suggest that involuntary or poorly prepared discharge may disrupt wellbeing perhaps because it reduces control over the timing and meaning of role exit, compresses the opportunity to prepare, and may sever military social ties before replacement relationships, roles and identities are established.

#### 3.3.3. Age and Identity Disruption

Reintegration difficulty and wellbeing were associated with age in two studies (Lee et al., 2020; Mitchell et al., 2020). This pattern should be interpreted cautiously given the small evidence base, but within those studies, younger veterans experienced more difficulties in the transition timeframe captured and lower life satisfaction than older veterans. Lee et al. (2020) also found that veterans aged between 20 and 40 reported stronger feelings of identity loss, with greater challenges among women and junior personnel, whereas veterans aged over 50 were less likely to report substantial identity loss.

#### 3.3.4. Military Identity Can Generate Social Isolation

Across the sampled studies, military identity was commonly noted to potentially generate social isolation, and feelings of being ‘othered’ or excluded from the civilian community. This theme was present in five studies (Brewster et al., 2021; Burkhart & Hogan, 2015; Flack & Kite, 2021; Keeling, 2024; Wakefield et al., 2024). Feelings of being socially isolated and not being able to connect with the civilian community had negative influences on veterans’ wellbeing and their view of their identity within the transition timeframes captured in these studies. In synthesising these study findings together to generate future research hypotheses, it may be plausible that social connections mediate the influence of military identity on veterans’ wellbeing. The contribution to wellbeing, however, may perhaps be determined by how strongly held the military identity is for the veteran. If it is held strongly and exclusively, veterans may have more difficulty maintaining or creating new social connections, which may adversely affect the veteran’s wellbeing. Transition timeframe variability in the sampled studies means the role of transition stages is unknown, but this would be worth examining longitudinally to understand how effects may unfold over time.

#### 3.3.5. Shared Identity and Social Connectivity

Within some of the included studies, veterans described being able to create connections with other veterans through their shared sense of identity, and while there may be circumstances where this can have negative influences on wellbeing, a shared veteran identity may also contribute to positive wellbeing gains. Two studies (Brewster et al., 2021; Waldhauser et al., 2021) reported that through this shared sense of identity, veterans were able to unlock support for each other, share resources and create social connections which positively contributed to veteran wellbeing. In Waldhauser et al.’s (2021) study evaluating a sports-based programme specifically for veterans, these connections were seen to extend beyond the programme, which demonstrated a potentially enduring wellbeing-contribution from creating spaces to allow veterans to connect. Brewster et al. (2021) found that when veterans connected with other veterans, positive implications for wellbeing were demonstrated; however, this connection may cause an interdependence on those relationships and a barrier to civilian life, therefore increasing feelings of exclusion from civilian communities and adversely influencing wellbeing.

#### 3.3.6. Gender and Identity Loss

Two studies identified the potential role that gendered experiences may play in veteran wellbeing (Burkhart & Hogan, 2015; Lee et al., 2020). Lee et al. (2020) highlighted women veterans are more likely to perceive loss of military identity as a challenge compared to men. This may be due to the unique service experiences that women undergo and the influence of socio-cultural gendered norms and expectations of women in the civilian community. Similarly, Burkhart and Hogan (2015) noted a sense of identity loss in their sample of women veterans, where the more masculine persona adopted while in a male-dominated military environment created identity issues returning to civilian life, in which perceived social norms may favour a less assertive or dominating approach in women.

#### 3.3.7. Serving in the Military Gives Life Meaning and Purpose

Across the included studies, nine reported findings related to meaning or purpose in life (see Table 3), with three containing longitudinal data (Gnall et al., 2023; Mitchell et al., 2020; Park et al., 2021), which enhances evidence trustworthiness. The studies sometimes referred to meaning and purpose interchangeably, hampering a clean separation between these related constructs. The synthesis distinguishes purpose from meaning in life where the included studies allowed this (e.g., where the Meaning in Life Questionnaire was used or where the term ‘purpose’ was used to refer to goal-oriented life direction). Across the sampled studies and within the transition timeframes captured, a common theme identified was that serving in the military gives life a sense of meaning, and in some studies, a sense of purpose. This theme was identified in five studies (Brewster et al., 2021; Carra et al., 2023; Mitchell et al., 2020; Park et al., 2021; Stevenson, 2020). Veterans often reported feeling unfulfilled after leaving the military and struggled to find their sense of meaning and purpose in new occupations. Where a strong sense of meaning in life remained post-transition, this was considered a potential protective factor (Park et al., 2021). Veterans who reported finding a sense of life meaning/purpose after transition also described reshaping their view of their self-identity in ways associated with positive wellbeing. By synthesising these study findings together to generate future hypotheses, it appears possible that a mechanistic relationship between military identity, life meaning/purpose and wellbeing may be worth exploring. Meaning/purpose in life may be one mechanism through which military identity is related to wellbeing for veterans. A less centralised military identity may lead to a veteran having an easier transition regarding reorienting their sense of life meaning/purpose, which in turn has the potential to positively influence veterans’ wellbeing. Alternatively, if the veteran is lacking life meaning/purpose, this may influence their ability to clearly define their new self-identity, creating negative influences on veterans’ wellbeing. More extensive longitudinal research beyond the longitudinal evidence represented in this theme (Mitchell et al., 2020; Park et al., 2021) is needed to unpack any potential causal mechanisms.

#### 3.3.8. Finding Life Meaning and Purpose After Service

Five of the included studies (Burkhart & Hogan, 2015; Carra et al., 2023; Gnall et al., 2023; Stevenson, 2020; Waldhauser et al., 2021) discussed how veterans searched for life meaning and purpose after service. Serving in the military was reported to provide a feeling of being a part of something bigger than the self and enabled veterans to feel that they have given back to their country, providing a sense of meaning to their military career and their life as a veteran. Finding life meaning and purpose after service was reported as important to veterans’ wellbeing within the transition periods captured in these studies. Longitudinally, meaning in life was associated with positive changes in veterans’ mental health QoL six months later (Gnall et al., 2023). Veterans who were able to find life meaning and purpose in new occupational activities such as employment opportunities, hobbies such as gardening or fishing, or community activities such as sports, experienced positive wellbeing. Veterans who chose to continue in service-related roles were able to reconnect with their sense of self, which increased their sense of safety and wellbeing. However, the results from Carra et al. (2023) indicated that there may be a risk of burnout if veterans continue in service-related roles.

#### 3.3.9. Group Memberships Give Meaning and Purpose

Group memberships, such as sporting groups or community groups, also appeared to give veterans opportunities to unlock psychological support and resources. Two studies (Wakefield et al., 2024; Waldhauser et al., 2021) reported the positive influence that group memberships can have on veteran wellbeing within the transition timeframes they investigated. If a veteran perceived the military had supported them through their transition, they were more likely to maintain group memberships or begin new ones (Wakefield et al., 2024). This group membership enabled veterans to create connections, which reportedly enhanced meaning in life, self-esteem and wellbeing. This study was complemented by Waldhauser et al.’s (2021) findings that through peer-connection and purposeful participation in group sport, veterans reported increased wellbeing, as indicated by the theme of ‘healthy body, healthy mind’.

#### 3.3.10. Summary of Thematic Synthesis

**Figure 2:**
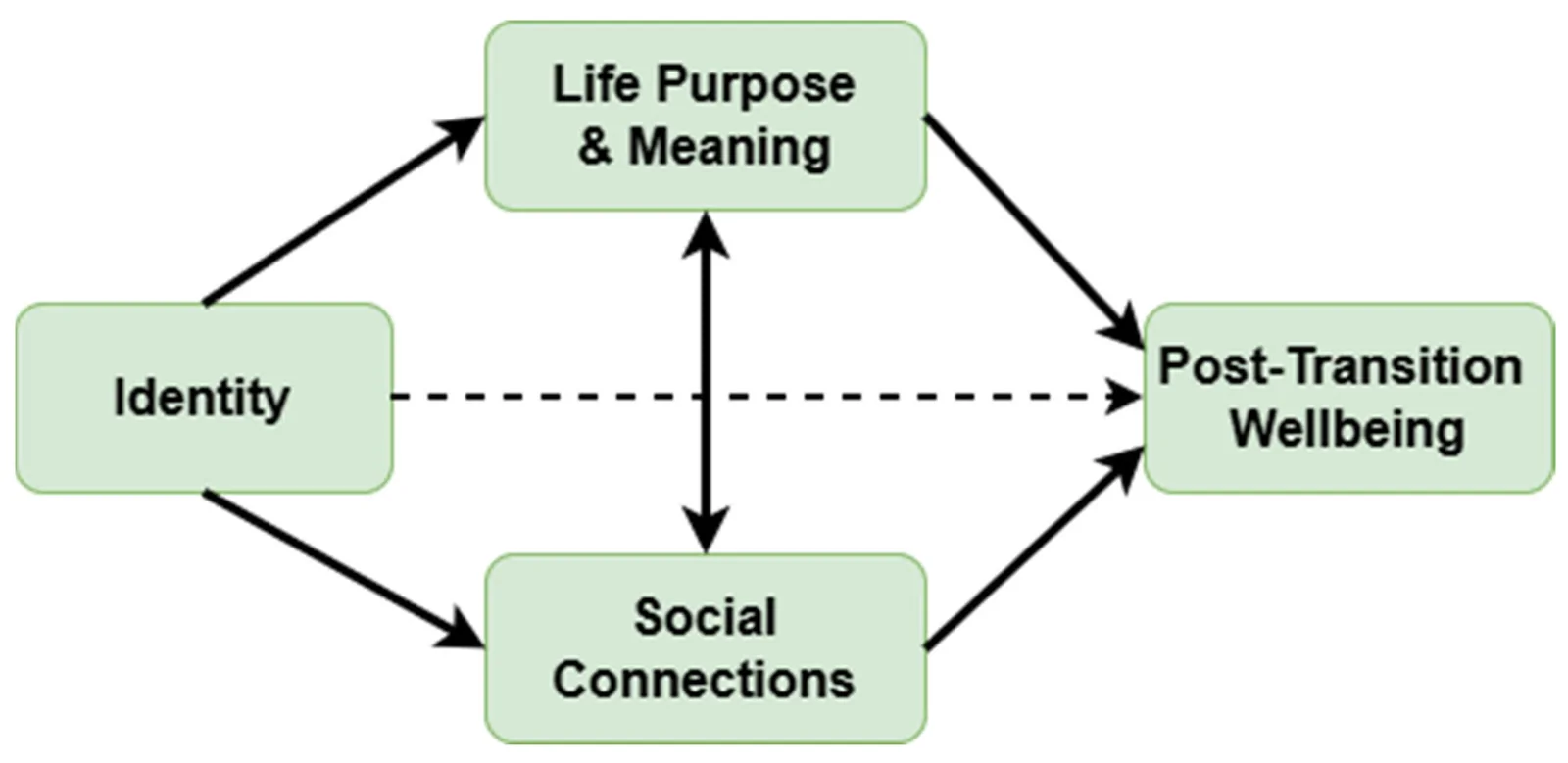
A hypothesis-generating conceptual model of candidate pathways to post-transition wellbeing for veterans. (Note. This is a conceptual model of candidate pathways linking military identity, life purpose/meaning, social connections and post-transition wellbeing. The proposed model is interpretive and hypothesis-generating, derived from the narrative synthesis, and does not represent a previously tested causal mediation model. The dotted line reflects the direct effect that identity may have on wellbeing, in addition to the indirect effects it may have through its influence on life meaning/purpose and social connections).

By drawing together the prominent themes of the narrative synthesis of evidence provided by the 15 included studies, we developed an interpretive conceptual model for the purpose of generating future research hypotheses. Figure 2 presents this proposed hypothesis-generating conceptual model, mapping possible pathways suggested by the evidence synthesis. The conceptual model is not a verified mediational model; rather, its purpose is to highlight potential candidate mechanisms and pathways that warrant empirical testing in future longitudinal and intervention research.

## 4. Discussion

### 4.1. Overview of Findings

This scoping review examined how veterans’ sense of identity, life meaning and purpose is associated with wellbeing during transition from military service. A small and heterogeneous evidence base of 15 studies was found in relation to this topic, indicating it is an emerging area in need of greater research attention. Evidence synthesis of the studies revealed two overarching themes and nine sub-themes relating to identity and life meaning/purpose. Taken together, the findings are most consistent with social identity approaches to transition (Haslam et al., 2021; Iyer et al., 2009), in which wellbeing depends not on shedding military identity altogether, but on whether it can be renegotiated alongside new roles, groups, and sources of meaning/purpose in life post-transition.

Whilst the existing evidence base was limited to 15 studies, the results consistently indicated that within the transition periods examined, military transition is associated with disruption to a veteran’s sense of identity and their sense of life meaning/purpose. Military service often fosters a strong collective identity, belonging, and camaraderie, all of which can be protective while serving. Difficulty appears to arise when military identity remains highly central after discharge but equivalent civilian roles, relationships, and sources of recognition are not yet available. In that context, veterans may experience loss, dislocation, and poorer wellbeing, especially when the military has been experienced as a primary source of belonging (Barnett et al., 2022; Flack & Kite, 2021). The age pattern identified in this review should also be treated cautiously because it was drawn from only two studies, albeit both suggested that younger veterans may face greater identity disruption during transition (Lee et al., 2020; Mitchell et al., 2020). Emerging evidence also suggests that women may face additional challenges where military and civilian gender expectations are in tension (Burkhart & Hogan, 2015; Lee et al., 2020).

Identity loss also appears to have social consequences post-discharge. In the included studies, feelings of exclusion, being ‘othered’, and a perceived civilian-military divide were associated with poorer wellbeing and more difficult adjustment (Brewster et al., 2021; Keeling, 2024; Waldhauser et al., 2021). Keeling’s (2024) narrative account of transition challenges linked greater transition strain to readiness, continued military values, and psychological challenges in negotiating identity after service. Veteran networks may therefore be both protective and limiting; they can provide support and understanding, but overreliance on them may slow broader community reintegration.

The study findings synthesised in this review indicate that military service can provide veterans with a sense of life meaning and purpose (e.g., Carra et al., 2023), often through service to something larger than the self. Life meaning and purpose after discharge appeared to vary depending on discharge circumstances. Veterans who medically or administratively transitioned reported a more disrupted sense of self than those who left voluntarily and, in some cases, felt their lives lacked value and direction (Barnett et al., 2022). Considered alongside the identity findings, this suggests that abrupt or non-volitional exits may interrupt not only employment plans but also the ongoing narrative through which service members understand who they are, what they contribute, and where they belong.

Evidence on the predictive role of meaning in life for wellbeing in veterans was consistent and supported by large longitudinal studies (Gnall et al., 2023; Mitchell et al., 2020; Park et al., 2021), although the transition timeframes varied. Veterans who reported successfully finding life meaning and purpose after service, particularly in service-related occupations, tended to report enhanced wellbeing (Carra et al., 2023). Maintaining or forming new group memberships post-transition was also associated with stronger meaning/purpose and with better wellbeing outcomes. Veterans who felt supported through transition were more likely to maintain or establish group memberships (Wakefield et al., 2024). This pattern again aligns with social identity explanations, suggesting that wellbeing may be supported when veterans can sustain valued connections while also building new ones.

### 4.2. Strengths and Limitations of the Scoping Review

This scoping review contributes to a developing body of review-level work on veteran transition by centering identity, life meaning/purpose, and wellbeing together. Strengths include the inclusion of qualitative and quantitative research and grey literature, allowing a broad mapping of an emerging field. Limitations include possible omission of relevant studies from additional databases, non-indexed sources, grey literature, and possible omission of recently published research due to the fast-moving nature of this area of research. A key limitation of the review is the capacity to only include articles that were written in English. Whilst studies conducted in non-English-speaking countries were eligible for inclusion, the final set of included studies were all conducted in English-speaking nations, meaning that the review findings may not be generalisable to non-English-speaking contexts. There was heterogeneity in study designs and sample sizes. Whilst study designs and their methodological trustworthiness were considered, formal critical appraisal of study quality was not conducted. This approach is consistent with scoping review design but limits conclusions about the certainty of the evidence. Therefore, the trustworthiness of the themes identified across the findings in the included studies should be interpreted with reference to the methodological strengths and limitations within the evidence base (reported in Supplementary Table S2). Conceptual boundary issues also affected study selection: wellbeing was operationalised using both explicit wellbeing measures and broader positively oriented psychosocial adjustment indicators, and some relevant qualitative narrative work contained themes focused more heavily on transition challenges than on explicit wellbeing outcomes. Although the inclusion of heterogenous wellbeing-related outcomes is appropriate within a scoping review methodology, this diversity should be considered when interpreting the overall synthesis of findings. Similarly, there was variation in how studies conceptualised life meaning and life purpose, which undermines interpretability. Finally, variation in transition timeframes warrants consideration, as some studies captured experiences within the early post-discharge period, whereas others reflected longer-term adjustment or did not report discharge timing. Thus, findings may not apply uniformly across all stages of civilian reintegration.

### 4.3. Gaps in the Evidence and Future Directions

Several knowledge gaps warrant attention. The lack of explicit definitions for key terms (identity, meaning, purpose, transition, veteran, and wellbeing) creates confusion in the evidence base and inconsistencies in the support provided to veterans. Clear, concise and explicit definitions should be established and articulated in all published research. The transition timeframe remains poorly defined in the literature, with limited research available in the first two years post-transition hindering understanding of how identity, purpose and wellbeing evolve during this acute phase of transition.

The traditional focus on mental illness may inadequately address veterans’ wellbeing needs. At the same time, this review also shows that the boundary between mental health and wellbeing is not always cleanly handled in the literature, reinforcing the need for clearer conceptualisation and reporting. Gender influences on transition experiences have received insufficient attention despite likely importance. The vast majority of participants in the reviewed studies were males, and one study explored how ‘sex’ shaped veteran wellbeing (Lee et al., 2020). A systematic review published after our review search period concluded that women’s transitions are shaped by gender discrimination, identity tensions, and difficulty accessing sex- and gender-sensitive services (Smith et al., 2025), suggesting this is a substantive gap rather than a marginal one.

While the emerging evidence indicates that identity, meaning and purpose in life matter for veteran wellbeing, the interplay between these factors remains underexplored. Several included studies already drew on theory, particularly social identity perspectives (Barnett et al., 2022; Wakefield et al., 2024). However, theory-use across the field remains inconsistent and is rarely translated into explicit mechanism testing. The hypothesis-generating conceptual model in Figure 2 therefore synthesises candidate pathways through which military identity may shape post-transition wellbeing directly and indirectly through life meaning/purpose and social connections. This conceptual model is speculative in nature, particularly given it was generated from an evidence synthesis of mostly cross-sectional studies (only three studies contained a longitudinal component). However, it highlights the need for future research that can uncover more information about temporal sequences and potential causal directionality using longitudinal and interventional designs. These future studies would benefit from examining the combined mechanisms through which continuity of valued group memberships, compatibility between military and civilian identities, and renewed life meaning and purpose influence subsequent post-transition wellbeing outcomes. These studies should be informed by multidimensional wellbeing theory, longitudinally test potential mediational mechanism pathways, and use more comprehensive assessment tools than the brief screening measures often used to date. Emerging research is beginning to address these gaps. For example, whilst not part of the evidence base included in this review, a new study by Grant et al. (2026) was published after our review search period, and found that subjective loss of past and future self were associated with poorer mental health and wellbeing in American and Australian veteran samples, with loss of future self emerging as the stronger predictor.

### 4.4. Implications and Recommendations

Given the emerging nature of the small and heterogeneous evidence base and the minimal representation of longitudinal data, only preliminary implications and recommendations can be offered. The findings from the reviewed studies provide initial suggestions regarding potentially fruitful avenues for transition programmes. In particular, identity and life meaning may be valuable transition targets alongside employment, housing, and mental ill-health. Practical improvements suggested by the review findings include consideration of earlier pre-discharge conversations about role exit and identity change, structured opportunities to translate military values and skills into civilian roles, facilitated access to both veteran and civilian groups, and follow-up support after discharge, especially for those leaving involuntarily.

The review findings do not necessarily suggest that military camaraderie or belonging should be weakened. Rather, the challenge relates to preserving the protective aspects of military connection while widening the person’s social world beyond the military. Programmes that bridge veterans into civilian communities, include families in transition planning, and create purposeful post-service roles may be particularly beneficial. The emerging literature on gender also suggests that supports may need to be more responsive to women’s transition experiences and to the possibility that military and civilian gender expectations might collide (Lee et al., 2020; Smith et al., 2025).

Finally, future research and programme design should build on the theoretical approaches already present in this literature. Studies such as Barnett et al. (2022) and Wakefield et al. (2024) indicate that social identity processes are central to transition, suggesting that interventions should not aim to strip away military identity, but help veterans integrate it with new identities, relationships, and sources of life meaning and purpose. Greater clarity about gender roles and identities, and transition timing will also be necessary if supports are to be responsive across different veteran groups.

## 5. Conclusions

This scoping review synthesised research into how veterans’ identity, life meaning and purpose are affected by transition and how these changes are related to wellbeing. Preliminary conclusions drawn from the emerging evidence suggest that serving in the military often provides a clear sense of life meaning/purpose, belonging, and identity. The findings indicate that transition out of the military is associated with disruption to these internal representations, particularly where discharge is non-volitional or where military identity remains highly central without alternative roles and relationships being established. Loss of identity and life meaning/purpose may contribute to isolation from civilian communities and poorer wellbeing. By mapping these candidate mechanisms, this review highlights significant opportunities for further research to be conducted to better understand pathways for fostering post-transition wellbeing, and points to the need for transition supports that attend not only to practical adjustment, but also to identity reconstruction, community connection, and renewed meaning and purpose in life.

## Figures and Tables

**Table 1:** Database search terms and inclusion/exclusion criteria.

	Inclusion Criteria [Key Search Terms]	Exclusion Criteria
Population	Veterans of the military (who are not serving in any capacity anymore) [Veteran OR ex-serving OR ex-defence OR ex-military]	Sample contains current serving military, including reservists. Sample includes a mix of serving and non-serving people without highlighting the difference and looking at each population separately for the outcomes. Sample contains civilians who have never served in the military.
Exposures	Personal feelings related to one’s identity and/or meaning/purpose in life [Purpose in life OR Identity OR Meaning in life OR Life purpose OR Social identity OR Belonging OR Social inclusion OR Meaningfulness OR Self Concept]	Personality/internal characteristics unrelated to life meaning/purpose or identity.
Outcomes	Positively oriented wellbeing-related outcomes indicative of positive psychosocial functioning/adjustment [Well being OR Wellbeing OR Well-being OR Quality of life OR QoL OR Happiness OR Life Satisfaction OR Satisfaction with Life OR Mental Health OR Positive outcome OR Wellness OR Flourish OR Satisfaction OR personal satisfaction OR Positive outcome]	Mental illness diagnosis only. Mental illness symptoms only. Deficit-oriented indicators of mental distress used only. Physical illness only. Physical symptoms only.
Types of Studies	Primary empirical research studies reporting the authors’ original research findings. Study designs may include qualitative, quantitative or mixed methods approaches. Published between 2000–Sept 2024. Published in the English language.	Secondary information sources, literature reviews, opinion pieces or editorial content published in journals. Published before the year 2000. Published in a language other than English.

Note. The sets of keywords listed under Population, Exposures, and Outcomes were combined using the Boolean operator ‘AND’.

**Table 2:** Included studies: concise summary of key characteristics.

Study	Sample Characteristics	Study Design	Key Constructs Examined	Main Contribution
Barnett et al. (2022)	Country: Australia n = 40 veterans (34 male gender) who had left the Defence Force in the past 5 years. Mean time since discharge was 3.33 years.	Qualitative	Identity and wellbeing were captured through interviews involving drawing a social identity map and a life-course map. Themes were derived related to identities and their contribution to transition wellbeing.	Derived themes indicated military life and the transition to civilian life influence veteran identity, the role of social groups/identity, and how values connected to military identity served as resources for veterans. They found that the context for discharge had implications for a veteran’s wellbeing and personal goals. Some elements of the military identity were potentially detrimental during civilian reintegration whereas other elements were assets. Within this sample, findings indicated that military values within veterans’ sense of self (e.g., strong sense of service and giving back to community) were reported as positive psychological resources for wellbeing within the transition timeframe captured.
Brewster et al. (2021)	Country: United Kingdom n = 30 veterans (27 male gender). Time since discharge not mentioned.	Qualitative	Identity was captured through the interview questions about their military experiences. Purpose, identity, and wellbeing represented in generated themes.	Being a veteran was reported by participants as something that gave life purpose in relation to others. But adopting the veteran identity had the risk of socially isolating themselves from civilians. Participant narratives highlighted key concepts such as fictive kinship (comrades like family) which influence veteran wellbeing, and how the visual culture of being recognised as a veteran had a positive effect on feelings of wellbeing and belonging.
Burkhart and Hogan (2015)	Country: United States n = 20 Veterans (all female gender). Time since discharge not mentioned.	Qualitative	Identity, meaning making and purpose during transition, as described in interviews. Categories/themes developed around coping with transition.	Participant narratives indicated that having pride in being a veteran-civilian and belonging to the veteran-civilian community helped to give meaning to their military career and was helpful for coping with the transition (although the transition timeframe was unclear).
Carra et al. (2023)	Country: Australia n = 10 veterans (9 male gender). Time since discharge was 2–7 years.	Qualitative	Life Meaning and Identity captured through the interview questions. Wellbeing through transition was represented in themes.	An overarching theme emerged around “filling the void” after military service through meaningful occupational participation. Participants described seeking meaning and fulfilment in civilian occupations, particularly those that allowed continuity of the mission to serve. These occupations were described as supporting connection, purpose, wellbeing and reconnection with self, while also helping participants develop a more positive view of civilian identity. The study also noted that continued service-related roles may carry a risk of burnout.
Gnall et al. (2023)	Country: United States. n = 367 veterans (218 male gender). Mean time since discharge was 3.4 years.	Quantitative 12-month longitudinal	Presence subscale from the Meaning in life Questionnaire. SF-12 Mental Composite score (higher scores indicate better mental QoL.)	The presence of meaning in life at baseline was significantly positively associated with mental health QoL 6 months later, and it was also associated with positive changes in mental health QoL. Large sample followed longitudinally enhances evidence trustworthiness.
Flack and Kite (2021)	Country: Australia n = 358 veterans (246 male gender). Discharged between 2000 and 2019 (65.8% were discharged between 2011 and 2019, 0–8 years since discharge).	Quantitative cross-sectional	Identities measured with Warrior Identity Scale. Social connectedness was also assessed. Subjective Wellbeing measured with WHOQOL-Bref (subscales of Physical QoL; Psychological QoL; Social QoL; and Environmental QoL)	Certain aspects of military identity were associated with either positive or negative wellbeing outcomes, with centrality of military identity and viewing the military as a family having a problematic influence, whereas high private and public regard for the military and less reliance on the military for social connection may be protective for wellbeing. Large sample size enhances generalisability of findings.
Keeling (2024)	Country: United States n = 6 veterans (all ‘male’) who had left the military between 1 and 12 years earlier (mean 5.3 years since discharge; only n = 1 was 12 years since discharge).	Qualitative	Identity and transition experiences were captured through veterans’ narratives, including accounts of transition challenges, readiness for transition, and continued military values. Psychosocial adjustment and transition challenges/adaptations were represented in themes.	Narrative analysis identified three master narratives: narratives of the challenges, narratives of readiness, and narratives of continued military values. The findings indicate that veterans’ transition experiences captured in this sample were shaped by practical readiness, psychological readiness for identity change, and the continuing role of military values. One sub-theme highlighted narratives about ‘meaningful and purposeful occupation’ and how this may relate to motivation, identity, wellbeing and psychosocial adjustment.
Lancaster et al. (2018)	Country: United States n = 1151 veterans (991 ‘males’). Time since discharge not mentioned.	Quantitative cross-sectional	Subscales of the Warrior Identity Scale were used, including identity exploration, identity commitment, public regard for the military, private regard for the military, centrality of military identity, viewing the military as a family, and connection with other military members. Subjective wellbeing assessed with the Satisfaction with Life Scale. Self-perceived postmilitary adjustment was assessed with two items.	Findings indicate military identity is related to important indicators of psychosocial functioning in military veterans. Those who reported high levels of personal regard for the military and high levels of public regard for the military indicated higher life satisfaction. Conversely, seeing the military as a family and identity exploration were associated with lower life satisfaction. Centrality was the only identity indicator not significantly associated with life satisfaction. Seeing the military as a family and identity exploration were both related to poorer self-perceived post-military adjustment (although the transition timeframe was unclear). Large representative sample enhances generalisability of findings.
Lee et al. (2020)	Country: Canada n = 1414 Veterans (85.8% male sex). All within 18 months of their release date.	Quantitative cross-sectional	Seven types of challenges of transitioning were measured with survey items, one of which was losing military identity. Self-perceived mental health was measured with a positively oriented 5-point Likert item (poor to excellent). Self-rated ease of post-military adjustment was measured with a single item.	All 7 perceived transition challenges were associated with greater odds of reporting post-military adjustment as difficult within the transition timeframe captured. The association was strongest for the loss of military identity. Those reporting good mental health had lower odds of reporting a loss of military identity. Females were more likely to perceive loss of military identity as a challenge. Large representative sample enhances the generalisability of findings.
Mitchell et al. (2020)	Country: United States n = 244 veteran sub-sample from a larger study of 1292 veterans (59.4% male sex). Mean time since ‘deployment’ was 6.2 years.	Mixed-methods: quantitative and qualitative baseline data; 3- and 6-month longitudinal quantitative data	Identity disruption was documented in expressive writing activities, and was coded yes if identity disruption was present and no if absent. Identity disruption included loss of meaning or purpose, disconnection between past/present/future selves, role disruption, and loss of self-worth. Subjective wellbeing was assessed with the Satisfaction with Life Scale at 3 time-points. Reintegration difficulty was also assessed with the Military to Civilian Questionnaire.	In their expressive writing, 49% of participants reported identity disruption. Those who reported identity disruption tended to report lower life satisfaction at baseline (negative association) and more reintegration difficulty (positive association). Satisfaction with life remained stable over the follow-up period. Large sample size followed longitudinally enhances evidence trustworthiness.
Park et al. (2021)	Country: United States n = 402 veterans (60% male gender). All had separated from the military within the last 5 years. Mean time between military discharge and baseline survey was 40.8 months.	Quantitative 12-month longitudinal	Presence subscale from the Meaning in life Questionnaire. SF-12 Mental Composite score (higher scores indicate better mental QoL.). Three surveys completed over 12 months.	Higher meaning in life was associated with greater mental QoL. Both meaning in life and mental QoL increased over the 12-month transition period captured. Meaning in life may be a protective factor against stressors experienced when undergoing transition, preventing mental health deterioration. Large sample size followed longitudinally enhances evidence trustworthiness.
Reed (2001)	Country: United Kingdom n = 121 veterans (all male gender). Mean time since leaving the military was 10.9 years.	Quantitative cross-sectional	Questionnaire items related to centrality of the ‘soldier’ or ‘civilian’ identities, and importance of the ‘soldier’ or ‘civilian’ identities. Continuity of identity (6-item scale). Subjective wellbeing assessed with the Satisfaction with Life Scale.	The identity distinctiveness acquired as a soldier in both social and group interaction and individual recognition were associated with more satisfaction with life, as was individually recognised self-esteem acquired as a soldier.
Stevenson (2020)	Country: United States n = 1 27-year-old Veteran, male gender. Time since discharge unclear.	Qualitative Case Study	Identity narratives and purpose narratives based on case notes. Therapist opinion on wellbeing and overall functioning.	In this single-case study, transition out of the military represented a loss of identity and limited purpose. A positive psychotherapy approach enabled the development of new self-identities and purpose to satisfy his needs for social connection, self-determination, satisfaction and overall wellbeing.
Wakefield et al. (2024)	Country: United Kingdom n = 210 Veterans (162 male gender), with subsample of n = 14 interviewed. Time since discharge not mentioned.	Mixed methods: (quantitative cross-sectional survey and qualitative interviews with subsample)	Meaning in life questionnaire short form, combined with personal control and self-esteem items to create a composite ‘psychological resources’ score. Exeter Identity Transition Scales (maintained and new group connections subscales). Wellbeing assessed with Satisfaction with Life Scale. Qualitative interview questions related to social psychological resources and wellbeing during veteran transition.	Maintaining group membership, new group membership, and having psychological resources (meaning, control and esteem) were all positively associated with greater satisfaction with life/wellbeing. Interview participants talked about lacking meaning after losing their military identity, and how group memberships boosted their sense of purpose, making them feel better and improving their wellbeing. Use of mixed methods enabled deeper probing of quantitative findings through qualitative interviews.
Waldhauser et al. (2021)	Country: Canada n = 12 veterans (all ‘male’). Time since discharge not mentioned.	Qualitative evaluation of an intervention	Shared military identity, social connection, healthy mind and body, and positive wellbeing were represented in themes.	Having a shared sense of identity supported a sense of social connectivity and was associated with positive mental wellbeing, healthy minds and bodies for male veterans.

**Table 3:** Descriptive and Analytical Themes Found From Synthesising the Included Studies.

Themes	Sub Themes	Studies with Findings Relevant to Sub Theme
Identity	Strong military identity—military is my family	(Barnett et al., 2022; Flack & Kite, 2021; Keeling, 2024; Lancaster et al., 2018);
	Mode of transition matters for identity	(Barnett et al., 2022; Keeling, 2024; Lee et al., 2020; Reed, 2001);
	Military identity can generate social isolation	(Brewster et al., 2021; Burkhart & Hogan, 2015; Flack & Kite, 2021; Keeling, 2024; Wakefield et al., 2024);
	Age and identity disruption	(Lee et al., 2020; Mitchell et al., 2020);
	Shared identity and social connectivity	(Brewster et al., 2021; Waldhauser et al., 2021);
	Gender and identity loss	(Burkhart & Hogan, 2015; Lee et al., 2020);
Meaning and Purpose in life	Serving in the military gives life meaning and purpose	(Brewster et al., 2021; Carra et al., 2023; Mitchell et al., 2020; Park et al., 2021; Stevenson, 2020);
	Finding life meaning and purpose after service	(Burkhart & Hogan, 2015; Carra et al., 2023; Gnall et al., 2023; Stevenson, 2020; Waldhauser et al., 2021);
	Group memberships give meaning and purpose	(Wakefield et al., 2024; Waldhauser et al., 2021);

## Data Availability

The original contributions presented in this scoping review study are included in the article/Supplementary Materials. Further inquiries can be directed to the corresponding author.

## Supplementary Materials

The following supporting information can be downloaded at: https://www.mdpi.com/article/10.3390/bs16081386/s1.

## References

[B1-behavsci-16-01386] Arksey H., O’Malley L. (2005). Scoping studies: Towards a methodological framework. International Journal of Social Research Methodology.

[B2-behavsci-16-01386] Barnett A., Savic M., Forbes D., Best D., Sandral E., Bathish R., Cheetham A., Lubman D. I. (2022). Transitioning to civilian life: The importance of social group engagement and identity among Australian defence force veterans. Australian & New Zealand Journal of Psychiatry.

[B3-behavsci-16-01386] Brewster L., McWade B., Clark S. J. A. (2021). A point of connection? Wellbeing, the veteran identity and older adults. Ageing & Society.

[B4-behavsci-16-01386] Bryant R., Lawrence-Wood E., Baur J., McFarlane A., Hodson S., Sadler N., Benassi H., Howell S., Abraham M., Iannos M., Hansen C., Searle A., Van Hooff M. (2019). Mental health changes over time: A longitudinal perspective: Mental health and wellbeing transition study.

[B5-behavsci-16-01386] Burkhart L., Hogan N. (2015). Being a female veteran: A grounded theory of coping with transitions. Social Work in Mental Health.

[B6-behavsci-16-01386] Carra K., Curtin M., Fortune T., Gordon B. (2023). Rural former service members participate in meaningful occupations to ‘fill the void’ after military service. Australian Occupational Therapy Journal.

[B7-behavsci-16-01386] Davies R. L., Cox S., Kelley M. L., Meca A., Milam A. L., Chae J. W. (2023). Posttraumatic stress disorder, personal identity, and meaning in life in U.S. veterans. Identity.

[B8-behavsci-16-01386] Department of Defence, Department of Veterans’ Affairs, Department of Health (2017). Australian Government response to the National mental health commission review into the suicide and self-harm prevention services available to current and former serving ADF members and their families.

[B9-behavsci-16-01386] Department of Veterans’ Affairs, Department of Defence (2018). Transition taskforce: Improving the transition experience.

[B10-behavsci-16-01386] Diener E., Emmons R. A., Larsen R. J., Griffin S. (1985). The satisfaction with life scale. Journal of Personality Assessment.

[B11-behavsci-16-01386] Dolan G., McCauley M., Murphy D. (2022). Factors influencing the salience of military/veteran identity post discharge: A scoping review. Journal of Veterans Studies.

[B12-behavsci-16-01386] Flack M., Kite L. (2021). Transition from military to civilian: Identity, social connectedness, and veteran wellbeing. PLoS ONE.

[B13-behavsci-16-01386] Gnall K. E., Sacco S. J., Park C. L., Mazure C. M., Hoff R. A. (2023). Life meaning and mental health in post-9/11 veterans: The mediating role of perceived stress. Anxiety, Stress, & Coping.

[B14-behavsci-16-01386] Grant C., Woodyatt L., Bowen H., Lane J. (2026). “Once a Soldier, always a Soldier” until you’re not: The effect of identity loss on mental health and well-being following military discharge. Military Psychology.

[B15-behavsci-16-01386] Haslam C., Haslam S. A., Jetten J., Cruwys T., Steffens N. K. (2021). Life change, social identity, and health. Annual Review of Psychology.

[B16-behavsci-16-01386] Heward C., Li W., Chun Tie Y., Waterworth P. (2024). A scoping review of military culture, military identity, and mental health outcomes in military personnel. Military Medicine.

[B17-behavsci-16-01386] Iasiello M., Ali K., van Agteren J., Kolovos E., Kyrios M., Kashdan T. B., Fassnacht D. B. (2024). What’s the difference between measures of wellbeing, quality of life, resilience, and coping? An umbrella review and concept map of 155 measures of positive mental health. International Journal of Wellbeing.

[B18-behavsci-16-01386] Iyer A., Jetten J., Tsivrikos D., Postmes T., Haslam S. A. (2009). The more (and the more compatible) the merrier: Multiple group memberships and identity compatibility as predictors of adjustment after life transitions. British Journal of Social Psychology.

[B19-behavsci-16-01386] Jarden A., Roache A. (2023). What is wellbeing?. International Journal of Environmental Research and Public Health.

[B20-behavsci-16-01386] Jones G. L., Hanley T. (2017). The psychological health and well-being experiences of female military veterans: A systematic review of the qualitative literature. Journal of the Royal Army Medical Corps.

[B21-behavsci-16-01386] Kaufman M. R., Eschliman E. L., Karver T. S. (2023). Differentiating sex and gender in health research to achieve gender equity. Bulletin of the World Health Organization.

[B22-behavsci-16-01386] Keeling M. (2024). Stories of transition: U.S. veterans’ narratives of transition to civilian life and the important role of identity. Journal of Military, Veteran and Family Health.

[B23-behavsci-16-01386] Kerr N. C., Lane S. J., Plotnikoff R. C., Ashby S. (2024). Occupational identity and the military to civilian transition of former serving Australian Defence Force members. Journal of Occupational Science.

[B24-behavsci-16-01386] Keyes C. L. M. (2002). The mental health continuum: From languishing to flourishing in life. Journal of Health and Social Behavior.

[B25-behavsci-16-01386] Lancaster S. L., Kintzle S., Castro C. A. (2018). Validation of the warrior identity scale in the Chicagoland veterans study. Identity.

[B26-behavsci-16-01386] Lawrence-Wood E., McFarlane A., Lawrence A., Sadler N., Hodson S., Benassi H., Bryant R., Korgaonkar M., Rosenfeld J., Sim M., Kelsall H., Abraham M., Baur J., Howell S., Hansen C., Iannos M., Searle A., Van Hooff M. (2019). Impact of combat report: Impact of combat study.

[B27-behavsci-16-01386] Lee J. E. C., Dursun S., Skomorovsky A., Thompson J. M. (2020). Correlates of perceived military to civilian transition challenges among Canadian Armed Forces Veterans. Journal of Military, Veteran and Family Health.

[B28-behavsci-16-01386] Martela F., Steger M. F. (2016). The three meanings of meaning in life: Distinguishing coherence, purpose, and significance. The Journal of Positive Psychology.

[B29-behavsci-16-01386] Mitchell L. L., Frazier P. A., Sayer N. A. (2020). Identity disruption and its association with mental health among veterans with reintegration difficulty. Developmental Psychology.

[B30-behavsci-16-01386] Moeck E. K., Takarangi M. K. T., Wadham B. (2022). Assessing student veterans’ academic outcomes and wellbeing: A scoping review. Journal of Veterans Studies.

[B31-behavsci-16-01386] Page M. J., McKenzie J. E., Bossuyt P. M., Boutron I., Hoffmann T. C., Mulrow C. D., Shamseer L., Tetzlaff J. M., Akl E. A., Brennan S. E., Chou R., Glanville J., Grimshaw J. M., Hróbjartsson A., Lalu M. M., Li T., Loder E. W., Mayo-Wilson E., McDonald S., Moher D. (2021). The PRISMA 2020 statement: An updated guideline for reporting systematic reviews. PLoS Medicine.

[B32-behavsci-16-01386] Park C. L., Sacco S. J., Finkelstein-Fox L., Sinnott S. M., Scoglio A. A. J., Lee S. Y., Gnall K. E., Mazure C., Shirk S. D., Hoff R. A., Kraus S. W. (2021). Post-9/11 military veterans’ adjustment to civilian life over time following separation from service. Journal of Clinical Psychology.

[B33-behavsci-16-01386] Pedlar D., Thompson J. M., Castro C. A., Castro C. A., Dursun S. (2019). Military-to-civilian transition theories and frameworks. Military veteran reintegration: Approach, management, and assessment of military veterans transitioning to civilian life.

[B34-behavsci-16-01386] Peters M. D. J., Godfrey C., McInerney P., Munn Z., Tricco A. C., Khalil H., Aromataris E., Lockwood C., Porritt K., Pilla B., Jordan Z. (2020). Scoping reviews. JBI manual for evidence synthesis.

[B35-behavsci-16-01386] Peters M. D. J., Godfrey C. M., Khalil H., McInerney P., Parker D., Soares C. B. (2015). Guidance for conducting systematic scoping reviews. International Journal of Evidence-Based Healthcare.

[B36-behavsci-16-01386] Pollock D., Evans C., Menghao Jia R., Alexander L., Pieper D., Brandão de Moraes É., Peters M. D. J., Tricco A. C., Khalil H., Godfrey C. M., Saran A., Campbell F., Munn Z. (2024). “How to”: Scoping review?. Journal of Clinical Epidemiology.

[B37-behavsci-16-01386] Reed A. (2001). A portfolio of academic, therapeutic practice and research work including an investigation of the potential threat to the identity of soldiers leaving the army and becoming civilians. Doctoral thesis.

[B38-behavsci-16-01386] Rodgers M., Sowden A., Petticrew M., Arai L., Roberts H., Britten N., Popay J. (2009). Testing methodological guidance on the conduct of narrative synthesis in systematic reviews: Effectiveness of interventions to promote smoke alarm ownership and function. Evaluation.

[B39-behavsci-16-01386] Royal Commission into Defence and Veteran Suicide (2024). Final report.

[B40-behavsci-16-01386] Sachdev S., Dixit S. (2024). Military to civilian cultural transition experiences of retired military personnel: A systematic meta-synthesis. Military Psychology.

[B41-behavsci-16-01386] Segal M. W. (2025). The military and the family as greedy institutions: Then and now. Armed Forces & Society.

[B42-behavsci-16-01386] Shepherd S., Sherman D. K., MacLean A., Kay A. C. (2021). The challenges of military veterans in their transition to the workplace: A call for integrating basic and applied psychological science. Perspectives on Psychological Science.

[B43-behavsci-16-01386] Shields D. M., Kuhl D., Lutz K., Frender J., Baumann N., Lopresti P. (2016). Mental health and well-being of military veterans during military to civilian transition: Review and analysis of the recent literature.

[B44-behavsci-16-01386] Smith A., Rafferty L., Croak B., Greenberg N., Khan R., Langston V., Sharp M.-L., Stagg A., Fear N., Stevelink S. (2025). A systematic review of military-to-civilian transition, the role of gender. PLoS ONE.

[B45-behavsci-16-01386] Snyder C. R., Lopez S. J. (2002). Handbook of positive psychology.

[B46-behavsci-16-01386] Steger M. F., Frazier P., Oishi S., Kaler M. (2006). The meaning in life questionnaire: Assessing the presence of and search for meaning in life. Journal of Counseling Psychology.

[B47-behavsci-16-01386] Stevenson B. J. (2020). Psychotherapy for veterans navigating the military-to-civilian transition: A case study. Journal of Clinical Psychology.

[B48-behavsci-16-01386] The WHOQOL Group (1998). Development of the World Health Organization WHOQOL-BREF quality of life assessment. Psychological Medicine.

[B49-behavsci-16-01386] Tkachuck M. A., Pavlacic J. M., Raley M. J., McCaslin S. E., Schulenberg S. E. (2022). Validating military culture: The factor analysis of a military-related adaptation of acculturation in veterans. Psychological Services.

[B50-behavsci-16-01386] Tricco A. C., Lillie E., Zarin W., O’Brien K. K., Colquhoun H., Levac D., Moher D., Peters M. D. J., Horsley T., Weeks L., Hempel S., Akl E. A., Chang C., McGowan J., Stewart L., Hartling L., Aldcroft A., Wilson M. G., Garritty C., Straus S. E. (2018). PRISMA extension for scoping reviews (PRISMA-ScR): Checklist and explanation. Annals of Internal Medicine.

[B51-behavsci-16-01386] U.S. Government Accountability Office [GAO] (2025). Military to civilian transition: Actions needed to ensure effective mental health screening at separation *(GAO-25-107205)*.

[B52-behavsci-16-01386] Van Hooff M., Lawrence-Wood E., Hodson S., Sadler N., Benassi H., Hansen C., Grace B., Avery J., Searle A., Iannos M., Abraham M., Baur J., McFarlane A. (2018). Mental health prevalence: Mental health and wellbeing transition study.

[B53-behavsci-16-01386] Wakefield J. R. H., Bowe M., Këllezi B., Haslam C., Bentley S. V., Milani Z., Gair H., McIntosh J. S. A. (2024). Brothers and sisters in arms: A mixed-methods investigation of the roles played by military support and social identity processes in the mental health of veterans during the transition to veterancy. Journal of Community & Applied Social Psychology.

[B54-behavsci-16-01386] Waldhauser K. J., O’Rourke J. J., Jackson B., Dimmock J. A., Beauchamp M. R. (2021). Purpose After Service through Sport (PASS): A social identity-informed program to support military veteran well-being. Sport, Exercise, and Performance Psychology.

[B55-behavsci-16-01386] Ware J. E., Kosinski M., Keller S. D. (1996). A 12-item short-form health survey: Construction of scales and preliminary tests of reliability and validity. Medical Care.

[B56-behavsci-16-01386] World Health Organization (2021). Health promotion glossary of terms 2021.

