# Supplementary material for: Transitioning to Civilian Life: How Are Life Meaning, Purpose and Identity Related to Wellbeing for Veterans? A Scoping Review
Source: Behav Sci (Basel). 2026 Aug 12;16(8):1386. doi: 10.3390/bs16081386 (PMC13509549; doi:10.3390/bs16081386)
Supplement: Supplementary file 1 [file behavsci-16-01386-s001.zip › behavsci-4356397-supplementary.pdf]

Supplementary Files

Supplementary Table S1

Structured Literature Database Search Strategy

**PsycINFO (on OVID)**		**CINAHL Complete (EBSCOhost)**		**Embase (Elsevier)**		**Medline (Elsevier)**	
**Stage**	**Keywords and *Subject Heading Terms***	**Stage**	**Keywords and *Subject Heading Terms***	**Stage**	**Keywords and *Subject Heading Terms***	**Stage**	**Keywords and *Subject Heading Terms***
**1**	Veteran	**1**	Veteran	**1**	Veteran	**1**	Veteran
**2**	*Military Veteran*	**2**	"Military veteran"	**2**	"Military veteran"	**2**	"Military veteran"
**3**	Ex-serving	**3**	Ex-serving	**3**	Ex-serving	**3**	Ex-serving
**4**	Ex-defence	**4**	Ex-defence	**4**	Ex-defence	**4**	Ex-defence
**5**	Ex-defense	**5**	Ex-defense	**5**	Ex-defense	**5**	Ex-defense
**6**	Ex-military	**6**	Ex-military	**6**	Ex-military	**6**	Ex-military
**7**	**#1 OR #2 OR #3 OR #4 OR #5 OR #6**	**7**	**#1 OR #2 OR #3 OR #4 OR #5 OR #6**	**7**	**#1 OR #2 OR #3 OR #4 OR #5 OR #6**	**7**	**#1 OR #2 OR #3 OR #4 OR #5 OR #6**
**8**	Army	**8**	Army	**8**	*Army*	**8**	*Army*
**9**	Navy	**9**	Navy	**9**	Navy	**9**	Navy
**10**	"Air Force"	**10**	"Air Force"	**10**	*"Air Force"*	**10**	*"Air Force"*
**11**	Airforce	**11**	Airforce	**11**	Airforce	**11**	Airforce
**12**	Military	**12**	Military	**12**	Military	**12**	Military
**13**	Soldier	**13**	Soldier	**13**	Soldier	**13**	Soldier
**14**	"Service Members"	**14**	"Service Members"	**14**	"Service Members"	**14**	"Service Members"
**15**	"Armed forces Personnel"	**15**	"Armed forces Personnel"	**15**	"Armed forces Personnel"	**15**	"Armed forces Personnel"
**16**	*"Military Personnel"*	**16**	*"Military Personnel"*	**16**	*"Military Personnel"*	**16**	*"Military Personnel"*

**17**	**# 8 OR #9 OR #10 OR #11 OR #12 OR # 13 OR #14 OR #15 OR #16**	**17**	**#8 OR #9 OR #10 OR #11 OR #12 OR # 13 OR #14 OR #15 OR #16**	**17**	**#8 OR #9 OR #10 OR #11 OR #12 OR # 13 OR #14 OR #15 OR #16**	**17**	**#8 OR #9 OR #10 OR #11 OR #12 OR # 13 OR #14 OR #15 OR #16**
**18**	Transition	**18**	Transition	**18**	Transition	**18**	Transition
**19**	Retir*	**19**	Retir*	**19**	Retir*	**19**	Retir*
**20**	"Military to civilian"	**20**	"Military to civilian"	**20**	"Military to civilian"	**20**	"Military to civilian"
**21**	Discharge	**21**	Discharge	**21**	Discharge	**21**	Discharge
**22**	Cessation	**22**	Cessation	**22**	Cessation	**22**	Cessation
**23**	Leaving	**23**	Leaving	**23**	Leaving	**23**	Leaving
**24**	Retirement	**24**	*Retirement*	**24**	Retirement	**24**	Retirement
**25**	**#18 OR #19 OR #20 OR #21 OR #22 OR #23 OR #24**	**25**	**#18 OR #19 OR #20 OR #21 OR #22 OR #23 OR #24**	**25**	**#18 OR #19 OR #20 OR #21 OR #22 OR #23 OR #24**	**25**	**#18 OR #19 OR #20 OR #21 OR #22 OR #23 OR #24**
**26**	**#17 AND #25**	**26**	**#17 AND #25**	**26**	**#17 AND #25**	**26**	**#17 AND #25**
**27**	**#7 OR #26**	**27**	**#7 OR #26**	**27**	**#7 OR #26**	**27**	**#7 OR #26**
**28**	"Purpose in life"	**28**	"Purpose in life"	**28**	"Purpose in life"	**28**	"Purpose in life"
**29**	Identity	**29**	Identity	**29**	Identity	**29**	Identity
**30**	"Meaning in life"	**30**	"Meaning in life"	**30**	"Meaning in life"	**30**	"Meaning in life"
**31**	belonging	**31**	belonging	**31**	belonging	**31**	belonging
**32**	"life purpose"	**32**	Meaningfulness	**32**	Meaningfulness	**32**	Meaningfulness
**33**	*"social identity"*	**33**	*"social inclusion"*	**33**	"social identity"	**33**	"social identity"
**34**	*"social inclusion"*	**34**	*"Life Purpose"*	**34**	"Life Purpose"	**34**	"Life Purpose"
**35**	*Meaningfulness*	**35**	*"Social Identity"*	**35**	"Social Inclusion"	**35**	"Social Inclusion"
**36**	*Self-concept*	**36**	Self-concept	**36**	Self-concept	**36**	Self-concept
**37**	**# 28 OR #29 OR #30 OR #31 OR #32 OR #33 OR #34 OR #35 OR #36**	**37**	**#28 OR #29 OR #30 OR #31 OR #32 OR #33 OR #34 OR #35 OR #36**	**37**	**#28 OR #29 OR #30 OR #31 OR #32 OR #33 OR #34 OR #35 OR #36**	**37**	**#28 OR #29 OR #30 OR #31 OR #32 OR #33 OR #34 OR #35 OR #36**
**38**	Wellbeing	**38**	Wellbeing	**38**	*Wellbeing*	**38**	*Wellbeing*

**39**	Well-being	**39**	Well-being	**39**	Well-being	**39**	Well-being
**40**	“Well being”	**40**	“Well being”	**40**	“Well being”	**40**	“Well being”
**41**	“quality of life”	**41**	“quality of life”	**41**	“quality of life”	**41**	“quality of life”
**42**	Happiness	**42**	Happiness	**42**	Happiness	**42**	Happiness
**43**	QOL	**43**	QOL	**43**	QOL	**43**	QOL
**44**	“life satisfaction”	**44**	“life satisfaction”	**44**	“life satisfaction”	**44**	“life satisfaction”
**45**	“satisfaction with life”	**45**	“satisfaction with life”	**45**	“satisfaction with life”	**45**	“satisfaction with life”
**46**	wellness	**46**	wellness	**46**	wellness	**46**	wellness
**47**	“mental health”	**47**	“mental health”	**47**	“mental health”	**47**	“mental health”
**48**	Flourish	**48**	Flourish	**48**	Flourish	**48**	Flourish
**49**	“positive outcome”	**49**	“positive outcome”	**49**	“positive outcome”	**49**	“positive outcome”
**50**	Satisfaction	**50**	Satisfaction	**50**	*Satisfaction*	**50**	*Satisfaction*
**51**	“personal satisfaction”	**51**	*“personal satisfaction”*	**51**	“personal satisfaction”	**51**	“personal satisfaction”
**52**	**#38 OR #39 OR #40 OR #41 OR #42 OR #43 OR #44 OR #45 OR #46 OR #47 OR #48 OR #49 OR #50 OR #51**	52	**#38 OR #39 OR #40 OR #41 OR #42 OR #43 OR #44 OR #45 OR #46 OR #47 OR #48 OR #49 OR #50 OR #51**	**52**	**#38 OR #39 OR #40 OR #41 OR #42 OR #43 OR #44 OR #45 OR #46 OR #47 OR #48 OR #49 OR #50 OR #51**	**52**	**#38 OR #39 OR #40 OR #41 OR #42 OR #43 OR #44 OR #45 OR #46 OR #47 OR #48 OR #49 OR #50 OR #51**
**53**	**#27 AND #37 AND #52**	**53**	**#27 AND #37 AND #52**	**53**	**#27 AND #37 AND #52**	**53**	**#27 AND #37 AND #52**

Supplementary Table S2

Characteristics of Included Studies

Author(s) (Year). Title.	Study Aims/Research Question(s)	Study Population	Study Design and Method	Exposure variables	Outcome Variables	Main Study Findings	Study Strengths/Limitations & Future Directions
Barnett et al. (2022). Transitioning to civilian life: The importance of social group engagement and identity among Australian Defence Force veterans.	"The study aims to examine how military identity and social group engagement influences veterans' transition." (p.1026)<br>Research Question: How do veterans social group engagements and identity influence their adjustment to civilian life and wellbeing?	n=40 participants who had left the Australian Defence Force (ADF) in the past 5 years (mean time since discharge was 3.33 years). The exclusion criteria were: (1) under 18 years old, (2) served as reservists prior to exiting the ADF or (3) experiencing psychological distress.<br>Gender: 6 Female participants and 34 males.<br>Mean age was 37 years old. All service types were represented with the breakdown by percentage as follows:<br>Army: 62.5%<br>Navy: 17.5%<br>Air Force:15%<br>Army and Air Force: 2.5%<br>Army and Navy: 2.5%<br>Length of service had a mean of 15.1 years.<br>The reasons for leaving defence were captured:<br>Discharge/resignation at own request: 60%<br>Medical Discharge: 27.5%<br>Compulsory age	Qualitative study through semi-structured in-depth interviews.<br>Participants were also asked to complete 2 mapping tasks, which provided a visual element to the interviews.<br>The interviews were coded using thematic analysis.	Identity- this was captured through the interview questions. The interview questions explored veterans' social group engagement and identity, life-course trajectories and wellbeing, and challenges faced when adjusting to civilian life.	Wellbeing – represented in the second mapping task which involved drawing a life course map. They drew a lifeline that showed ups and downs which represented wellbeing from 1 year prior to joining the ADF to the present, labels were included to represent the ups and downs.	The results of the interviews were presented as 3 themes: a) how military life and that transition to civilian life influence veteran identity; b) social groups and identity during the transition; and c) how value connected to military identity served as resources for veterans after discharge.<br>Their findings suggest that the stronger the military identity, the more likely that a veteran will have negative impacts on their wellbeing post transition. For example, those that were ambivalent about their military identity did not experience negative impacts to their wellbeing, however if their service was terminated through medical or administrative discharge and ended abruptly this was often associated with negative impacts on veteran wellbeing. They found that the context for discharge was an important factor on the implications to a veteran's wellbeing, as well as life stages and personal goals. (p.1032). They also found that military values within veterans' sense of self (such as a strong sense of service, altruism and giving back to community) were positive psychological resources for wellbeing through transition. Thus, some elements of the military	Self-selecting sample who participated may not be relevant to other veterans. Sample was Australian Veterans. Other countries may differ, along with supports that are offered in other jurisdictions.<br><br>Future Research needed to expand this research into other countries.

Author(s) (Year). Title.	Study Aims/Research Question(s)	Study Population	Study Design and Method	Exposure variables	Outcome Variables	Main Study Findings	Study Strengths/Limitations & Future Directions
		retirement: 2.5%<br>Other:10%<br><br>Country: Australia				identity were assets, but others were potentially detrimental during the civilian reintegration process. Veterans' reintegration into civilian life may be improved through better consideration how pre-existing, supportive civilian social ties can be maintained through their careers. This could be further enhanced through linkage to volunteer programs, and ex-service support organisations which may in turn support veterans' engagement with identities other than military and increase social ties.	
Brewster et al. (2021). A point of connection? Wellbeing, the veteran identity of older adults.	"The study aims to identify the specific challenges that ex-service personnel may have, report findings from a qualitative study focused on how older veterans told stories of military service and what those stories revealed about wellbeing"<br><br>Research Question: Older veterans were asked to explore their military experiences, with questions framed around comradeship, returning home, and veteran identity to understand how	n=30 veterans. Gender: 27 males and 3 females. The participants had to be born prior to 1 January 1950 and length of service ranged from 2-30+ years. The type of enlistment was captured from second world war conscription, national service and voluntary. All services were represented.<br><br>Time since discharge not mentioned.<br><br>Country: United Kingdom.	Qualitative study. Analysis was conducted using a data-driven constant comparison approach to foreground experience, using themes developed from the data and where appropriate from the literature as a sensitising framework.	Identity- was captured though the interview questions about their military experiences. Interview questions were intentionally broad and focused on predominantly positive or neutral experiences.	The results of the interviews were presented as themes examining the ways that wellbeing and social connection were affected by the status of being a veteran. Three themes were found:<br>1- loneliness and social isolation<br>2- Fictive Kinship<br>3- Military visual culture	Being a veteran was something that gave life meaning and purpose in relation to others. However, by adopting the veteran identity had the risk of isolating themselves from the civilian population, in that they 'othered' themselves and constructed an identity that was separate from the civilian community. This identity could be deemed as exceptional to some veterans, further constructing a barrier between veterans and people who have not served in the military which increased social isolation. This identity created distinctive challenges for veterans which were both exacerbated and mitigated by the adoption of the veteran identity. By adopting the veteran identity, they were able to find points of connections with other veterans which allowed them to access each other as a resource for sharing and	The research identified that veterans were not involved as co researchers, which could have had implication on the impact of sharing. The gathering of participants was a self-selecting group, it could not be certain that this group accurately represents wider community views. The paper did not go into the different identities, such as differences between male and female experiences. The research project engaged with a wide group of older adults across the UK which included rural communities and in major suburban areas. This diverse group enabled exploration of the role adopting a veteran identity had in a

Author(s) (Year). Title.	Study Aims/Research Question(s)	Study Population	Study Design and Method	Exposure variables	Outcome Variables	Main Study Findings	Study Strengths/Limitations & Future Directions
	narrating experiences affects wellbeing.					connection and belonging however it created feelings of exclusion from the civilian community. Participant narratives highlighted key concepts such as fictive kinship (comrades like family) which influence veteran wellbeing, and how the visual culture of being recognised as a veteran had a positive effect on feelings of wellbeing and belonging.	community formation. Future Research: The study suggested future research to be conducted to address these limitations, plus research into how rejection of the veteran identity has on wellbeing.
Burkhart & Hogan (2015). Being a female veteran: A grounded theory of coping with transitions.	"The purpose of this grounded theory study is to discover the categories and processed grounded in the experience of female veterans who transitioned into, through and out of the military" (p.111).	n=20 Veterans, all female gender, 8 whom served in non-health care settings. Participants served from 2-30 years with a mean of 16.7 years. The mean age was 45 with a range between 23-65 years of age. Time since discharge not mentioned.<br><br>Country: United States of America	Qualitative study design using grounded theory methods.<br><br>Participants participated in telephone interviews (n=10) or in-person interviews (n=10). Interviews lasted 2 hours, and were scripted to elicit experiences transitioning into, though, and out of the military. Saturation was reached at 17 interviews with an additional 3 conducted to ensure data saturation.	Identity, meaning making and purpose, as described in interviews.	The findings from the interviews were presented as categories, with the core category being 'coping with transitions.<br>(O1) Challenges of transitioning<br>7 categories identified:<br>1- Choosing the military<br>2- Adapting to the military<br>3- Being in the military<br>4- Being a female in the military<br>5- Departing the military<br>6- Experiencing stressors of being a civilian<br>7- Making meaning of being a veteran-civilian	Category 6 and 7 both highlighted challenges of identity and loss of belonging for veterans. These findings highlighted that civilian and military sectors are widely separate, following different social and psychological rules and bridging the two worlds is difficult and caused impacts on a veteran's sense of identity and belonging. The only way participants could maintain their identity within the civilian sector was to re-establish connections with the military, friends from the military and veteran groups.<br>"Making Meaning of Being a Veteran-Civilian. Many participants described giving meaning to their military career as a time of reflection and reconnection to military values as helpful to cope with this transition" (p.120). This included two subcategories: having pride in being a veteran-civilian and belonging to the veteran-civilian community.	Strengths: The research provides a context to better understand the unique experiences and needs of female veterans to better assist in transition.<br><br>Limitations: The study focused solely on women veteran's transition. It did not include male experiences or perspectives. All the participants also entered the military during adolescence but re-entered civilian life at different times in their lives. The transition timeframe was unclear.

Author(s) (Year). Title.	Study Aims/Research Question(s)	Study Population	Study Design and Method	Exposure variables	Outcome Variables	Main Study Findings	Study Strengths/Limitations & Future Directions
Carra et al. (2023). Rural former service members participate in meaningful occupations to 'fill the void' after military service.	"The aim of the study was to explore rural former Australian Defence Force members experiences of participating in occupations and how these experiences influenced their health and adjustment'.<br><br>Gap statement: research is needed to understand how participating in everyday occupations influence health and adjustment during the transition from military service, particularly in rural areas.	n=10 Rural Veterans. Gender: 1 female and 9 males, who had either served in the Army, Navy or Air Force. Ages ranged from between 20-59 years of age, with length of service between 2-more than 20 years. The time since discharge was between 2-7 years and type of discharge was captured with 6 medical discharge and 4 voluntary.<br><br>Country: Australia	Qualitative study through semi-structured videoconferencing interviews. 9 of the participants conducted a second interview, in which summaries were provided to each participant to further clarify the information captured and prompt further discussion surrounding emerging themes. Data was coded into themes using Interpretative Phenomenological Analysis (IPA).	Meaning in life (purpose through meaningful occupational participation) was captured though the interview questions. Identity was captured through the interview questions. Open-ended questions were asked in interviews to prompt participants to reflect on their transition period.	The findings from the interviews were presented as themes (in which wellbeing was represented). One overarching theme detected: 'It can't all be for nothing': searching for ways to fill the void through meaningful occupation<br><br>2 sub- themes within this:<br>1- You can't heal if you're still in a battlefield: participating in occupations to reestablish safety and wellbeing<br>2- I was in uniform, and I could still do well: participating in occupations to reconnect with self and continue the mission to serve	An overarching theme emerged from the interviews, 'it can't all be for nothing'- searching for ways to fill the void through meaningful occupational participation. Central to this theme was the deep 'void' sensed after ending a military career, and a desire to find meaning and fulfilment in civilian life through their chosen occupations. Participants searched for this meaning and fulfilment in civilian occupations. Two sub-themes captured the ways participants filled the void after leaving the military: 'You can't heal if you are still in a battlefield: participating in occupations to re-establish safety and wellbeing' and 'participating in occupations to reconnect with self and continue the mission to serve'. Continuity in service-related roles enabled participants to reconnect with themselves and their life mission to serve others. This continuation of service and structure supported participants sense of safety and wellbeing moving through their transition. By finding meaning in their occupations, participants were able to increase their wellbeing and meaning and reconstruct a positive view of being a civilian. Finding connection and a new sense of purpose in civilian life was critical to participants wellbeing and search for meaning. However, there is a risk to returning to service-related occupations as this can increase burnout, and	Limitations: Study size and demographic, only 1 female participated in the research. Recruitment was limited by the COVID-19 pandemic, and the conduct of the interviews could not occur face-to-face.<br>Future research: Exploration of the impacts of a wider and more varied demographic, with varied ages, branch of service and discharge type. Further research would also be beneficial to be conducted face to face to enable more in-depth exploration of experiences. Mixed methods research could also enhance the area of study in the future.

Author(s) (Year). Title.	Study Aims/Research Question(s)	Study Population	Study Design and Method	Exposure variables	Outcome Variables	Main Study Findings	Study Strengths/Limitations & Future Directions
						adversely impact wellbeing. Engaging in occupations that enable connection, purpose and meaning positively impacted veterans' wellbeing and enabled them to shape a new perspective on being a civilian.	
Gnall et al. (2023). Life meaning and mental health in post-9/11 veterans: the mediating role of perceived stress.	"The present study sought to empirically test this theoretical pathway linking meaning in life to stress, and stress to dimensions of mental health, in a sample of post-9/11 veterans surveyed an average of 3-4 years post deployment".	n=367 participants. Average time since discharge was 3.4 years and average age was 36 years. Gender: 59.4% men (n=218) and 40.6% women (n=149). Men had a lower meaning of life baseline compared to women. Majority of participants were white (80.1%), with a racial minority status at 19.9%.<br><br>Country: United States of America	Quantitative study which was a secondary analysis of the Survey of Experiences of Returning Veterans (SERV), a 12-month longitudinal observational study. Participants were interviewed at 5 different timepoints, (baseline and then 3, 6, 9 and 12 months) using structured telephone interviews. Participants who completed the baseline, 6-month and 12-month follow up interviews were examined in this study.	Meaning in life- Measured by the self-report meaning in life questionnaire (MLQ, Steger et al). The 5-tiem subscale 'presence of meaning in life' was used in the study.<br>Perceived stress was another predictor variable in this study.	Mental health quality of life (QoL) measured over the past month measured via the Mental Component Score of the Short Form Health Survey (SF 12, Ware et al., 1996). Higher scores mean more greater mental health QoL.<br>Other Outcome variables were depression, stress, PTSD, anxiety, and insomnia symptoms.	The presence of meaning in life at baseline was significantly positively associated with mental health QoL 6-months later, and it was also associated with positive changes in mental health QoL from 6-to-12 months in path models. These path models also showed that perceived stress mediated the association between these variables, i.e., the positive impact of life-meaning on improving mental health QoL occurred through its link with lower perceived stress. Meaning in life at baseline was negatively (and strongly) associated with stress at baseline and all dimensions of mental health at 6 months (PTSD, depression, anxiety, insomnia).	Large longitudinal study tracking veteran outcomes over time, enhancing evidence trustworthiness, although it was a convenience sample. Female veterans were oversampled to enhance generalisability to the changing demographic of veterans. However, the sample had been out of service for a number of years, the study does not involve an intervention, examination over a longer period of time may be required to reveal more significant changes across dimensions of mental wellbeing. Longer time frame to capture changes may be of use for future research to determine other changes across wellbeing dimensions.
Flack & Kite (2021). Transition from military to civilian: identity,	Flack & Kite (2021) identified a knowledge gap regarding how military identity and	n=358 contemporary ex-serving Australian Defence Force veterans who had discharged from the ADF between 2000-	Cross-sectional quantitative study. Convenience sample recruited for	Identity- measured using the Warrior Identity Scale (WIS). This scale was developed as a	Subjective Wellbeing – measured through the WHO quality of life Bref (WHOQOL-Bref). This is a short	'Family military identity' and 'Private military identity' exerted a direct influence on psychological, environmental and physical wellbeing. Public, connect,	Large sample size enhances generalisability of findings.<br><br>Limitations:<br>The cross-sectional design

Author(s) (Year). Title.	Study Aims/Research Question(s)	Study Population	Study Design and Method	Exposure variables	Outcome Variables	Main Study Findings	Study Strengths/Limitations & Future Directions
social connectedness, and veteran wellbeing.	social connectedness interrelate to influence contemporary veteran wellbeing following transition to civilian life. They aimed to explore the potential moderating and mediating effects that social connectedness has in the relationship between military identity and wellbeing.	2019. 65.8 percent of the participants discharged between 2011 and 2019, 0-8 search since discharge. Average service time of 14 years. 1.7% identified as Indigenous Australians, 88.5% of Western European decent and 9.8% from other backgrounds.<br>Ages ranged from 20-66 years or older with a mean age of 42 years.<br>Gender: 111 females were included in the survey and 246 males. One of the participants did not disclose their gender.<br>The sample included veterans from:<br>Australian Army: 69.8%<br>Royal Australian Navy: 18.7%<br>Royal Australian Air Force: 8.9%<br>With 2.5% reporting serving in one of more branches.<br><br>Country: Australia	anonymous online survey.	multidimensional measure of military identity. The WIS was still under development at the time of the study, and a final scale of 20 items from the initial pool of 66 items was generated via factor analysis. The final 20 items reflected the 6 subscale domains of military identity referred to as 'private', 'public', 'connect', 'interdependence', 'family', and 'centrality'. Scores are scaled in a positive direction, with higher scores indicating a higher degree on the subscale.<br>Social connectedness was also an exposure variable in the study.	26 item scale adapted from the WHOQOL-100. Generates 4 QoL scores: Physical QoL; Psychological QoL; Social QoL; and Environmental QoL Domain scores are transformed to generate a 0-100 scale for each domain, with higher scores indicating greater subjective QoL.<br>Psychological distress was also an outcome variable in the study.	interdependence, and centrality identity subscales had an indirect effect on all 4 wellbeing outcomes through their influence on social connectedness (i.e., social connectedness mediated their associations with wellbeing).<br>Certain aspects of military identity had either positive or negative impacts on wellbeing outcomes, with centrality of military identity and viewing the military as a family having a problematic impact, whereas high private and public regard for the military and less reliance on the military for social connection may be protective for wellbeing. Protective effects of a perceived connectedness to former military networks and negative effects of a centrality of military identity on wellbeing in an Australian military sample.<br>(E1) An ensuing military identity in conjunction with disconnect from civilian society may interrelate to influence (O) wellbeing.<br>The study highlights the protective and inhibiting effects of military identity to both social connectedness and wellbeing that may otherwise be obfuscated by a unidimensional focus on military identity.	means causal pathways cannot be determined. Convenience sampling method may have introduced sampling bias. Survey self-report bias. Therefore, potentially limiting the conclusion made regarding the WIS and path analysis findings to other populations.<br><br>Future directions<br>Future research could examine problematic and protective profiles via cluster analysis in both clinical and non-clinical populations. The approach may further inform veteran centric intervention programs aimed at improving the individual wellbeing of contemporary veterans.<br>Future interventions that consider the different aspects of military identity whilst promoting a broader social network may be of benefit.
Keeling (2024). Stories of transition: U.S. Veterans' narratives of	The study aimed to gain a deep understanding of veterans' transition to civilian life, the	n=6 veterans reported as 'male' (sex vs gender unspecified). All residing in the Chicagoland area who had left the military	Qualitative narrative study. Veterans were interviewed using a narrative approach so they could share their	Identity and transition experiences were captured through veterans' narratives, including accounts of	Psychosocial adjustment and transition challenges. Narrative themes focus on identity,	Narrative analysis identified three master narratives: narratives of the challenges, narratives of readiness, and narratives of continued military values. Military identity and values	Strengths: Provides rich qualitative insight into veterans' transition stories and the role of identity in adjustment.

Author(s) (Year). Title.	Study Aims/Research Question(s)	Study Population	Study Design and Method	Exposure variables	Outcome Variables	Main Study Findings	Study Strengths/Limitations & Future Directions
transition to civilian life and the important role of identity.	challenges they face, and the adaptive and maladaptive ways in which they manage those challenges.	between 1 and 12 years earlier. (average of 5.3 years; only n=1 who had left 12 years prior).<br><br>Country: United States of America	transition stories. Narrative analysis was used to identify master narratives across participants' accounts.	transition challenges, readiness for transition, and continued military values.	psychological readiness, psychological challenge, and how they affect wellbeing.	continue to shape post-service adjustment, preparation for transition matters, and transition can involve psychological challenge and possible existential crisis when identity is disrupted. The findings indicate that veterans' transition experiences as captured in this sample are shaped not only by practical readiness but also by the positive impact of psychological readiness for identity change. One sub-theme highlighted narratives about 'meaningful and purposeful occupation' and how this may relate to motivation, identity, wellbeing and psychosocial adjustment. The study provides qualitative insight into how identity, meaning and purpose may relate to psychosocial adjustment during transition, although wellbeing was not measured directly.	Limitations: Small all-male United States sample drawn from one geographic area; findings may not generalise to women veterans or broader veteran populations. The study did not use a formal wellbeing measure, so its relevance is to broader psychosocial adjustment and identity-related wellbeing rather than measured wellbeing.<br><br>Future research: Further research should examine identity-focused transition support in larger and more diverse veteran samples, including longitudinal work that follows identity change over time.
Lancaster et al. (2018). Validation of the Warrior Identity Scale in the Chicagoland Veterans Study	"The aim of the study was to examine the factor structure and convergent validity of the WIS in a large sample of military veterans". This included examining the association of military identity on psychosocial functioning in veterans.	n=1151 Participants from the Chicagoland Veterans Study. Sample included 160 'females' (13.9%) and 991 'males' (86.1%) (sex vs gender unspecified). All had served in the Army, Navy, Marine corps, Air force or Coast guard. The mean age was 52.68 with a mean length of service being 7.1 years. Time since discharge not mentioned.	Quantitative cross-sectional study using self-report surveys.	Identity measured with the WIS (Warrior Identity Scale) – A revised shortened scale was adopted for this study, 34 items instead of 60. It contains 7 subscales including identity exploration, identity commitment, public regard for the military, private regard for the military, centrality of military identity, viewing the military	Subjective wellbeing measured with the 5-item Satisfaction with Life Scale (Diener et al). Other outcome variables were PTSD, depression, suicide behaviours, somatic symptoms, and perceived postmilitary adjustment.	Most dimensions of identity increase as the number of years served and number of deployments increases. Seeing the military as a family and identity exploration were both related to poor postmilitary adjustment. Those who reported high levels of personal regard for the military and high levels of public regard for the military indicated less distress and higher life satisfaction. Conversely, seeing the military as a family and identity exploration were associated with increased levels of distress and lower life satisfaction. Identity	The study was strengthened by the large sample representative of the U.S military populations. This was further strengthened by the large range of outcomes that helped elucidate the differences between domains of identity.<br><br>Limitations: Reliance on self-reporting measures and a cross-sectional methodology. Time since discharge unclear. Possible

Author(s) (Year). Title.	Study Aims/Research Question(s)	Study Population	Study Design and Method	Exposure variables	Outcome Variables	Main Study Findings	Study Strengths/Limitations & Future Directions
		Country: United States of America		as a family, and connection with other military members.		commitment and centrality of identity only weakly predicted the indicators of distress, which may suggest that these dimensions are less important in a clinical context. Centrality was the only identity indicator not significantly associated with life satisfaction. Military identity should be viewed as a multidimensional construct. Military identity is related to important indicators of psychosocial functioning in military veterans.	cultural differences in identity.
Lee et al. (2020). Correlates of perceived military to civilian transition challenges among Canadian Armed Forces veterans.	"The goal was to provide a clearer understanding of military to civilian transition challenges in order to inform planning for services supporting the well-being of Canadian Armed Forces (CAF) members at this important stage of life".<br><br>Gap statement: More detail about the population wide extent and correlates of transition challenges is needed to inform the development of relevant support services and programs.	n=1414 veterans. All surveyed within 18 months of their release date, with a 75% response rate. All participants had to have served a minimum of 2 years and veterans who were released for misconduct or unsatisfactory service were excluded (due to the challenges in accessing that group).<br><br>49.6% of veterans were medically released, 50.4% released through non-medical categories. Sample sex reported as primarily male (85.8%), and aged between 30-49 years of age and majority had served 20 years or more.	Quantitative cross-sectional study design, participants responded to a series of survey questions- questions were not provided.<br><br>A stratified random sample was drawn from the CAF database.	Challenges of transitioning were measured with 10 items on which participants rated the level of challenge they experienced. Pairs of similar items were combined, and a final set of scores for 7 types of challenges were generated.<br>7 perceived challenges:<br>1- Being financially prepared<br>2- Finding employment<br>3- Finding educational opportunities<br>4- Relocating<br>5- Finding a health practitioner<br>6- Understanding benefits<br>7- Losing military	Perceived Mental Health was measured through self-rating on a 5-point Likert-type scale (1=poor, 2=fair, 3=good, 4=very good, 5=excellent). Perceived physical health was also measured in the same manner.<br><br>Self-rated ease of post-military adjustment was measured with a single item rated from 1= very easy to 5= very difficult.	All 7 perceived transition challenges were associated with difficulty in post military adjustment (lower mental health and physical health); however, the association was strongest for the loss of military identity. Those reporting good mental health had lower odds of reporting a loss of military identity. Veterans who were medically released or reported poor physical and mental health had substantially greater odds of perceiving a loss of military identity. Regarding demographic and military characteristics, the odds of perceiving loss of military identity as challenging were highest among veterans aged between 30-39 years, those who were medically released, female and junior soldiers as well as those who had primarily served in the army, had deployed in Afghanistan and served between 6-9 years.<br>Conversely veterans who were older	Strengths: Used a large representative sample of newly released CAF veterans, with a high response rate to the survey. Clear reporting of time since discharge.<br><br>Limitations: Due to the cross-sectional nature no causal relationships can be inferred. Only single item measures of self-rated physical and mental health were examined. Study excluded veterans discharged for misconduct or unsatisfactory service.<br><br>Implications: This study supports recommendations that social identity challenges should be addressed during military transition. Additional research on issues

Author(s) (Year). Title.	Study Aims/Research Question(s)	Study Population	Study Design and Method	Exposure variables	Outcome Variables	Main Study Findings	Study Strengths/Limitations & Future Directions
	The objective of the Canadian Armed Forces Transition and Wellbeing Survey (CAFTWS) were to:<br>1- Identify aspects of the military to civilian transition that are perceived as challenging by CAF veterans<br>2- Explore the association of type of release and health status with commonly perceived transition challenges.	Country: Canada		identity.<br>Subsequent analyses focussed primarily on loss of military identity.		than 50 and had been deployed demonstrated lower odds of perceiving a loss of military identity.<br>Women were more likely to perceive loss of military identity as a challenge. This may be due to several unique aspects of women's military careers. A strong military identity for a female may make the adjustment to civilian culture more difficult due to the cultural norms and expectations of what it is to be a civilian female.	surrounding social identity during military to civilian transition might include further development of relevant measures and the evaluation approaches for addressing such challenges.
Mitchell et al. (2020). Identity disruption and its association with mental health among veterans with reintegration difficulty.	"This is the first study to our knowledge that quantitatively assessed the relations between veterans' identity and mental health constructs."<br>Research Hypothesis: Veterans reporting identity disruption would report lower levels of social support and satisfaction with life and higher levels of PTSD symptom severity and reintegration difficulty at baseline.	The major study included n=1292 veterans from the 'military to civilian study' who were randomised into three study conditions: expressive writing (n=508), control writing (n=507), or no writing control (n=207).<br>This sub-study was based on the sample of n=244 participants who completed four expressive writing sessions. The sample reported sex and included 59.4% male and 40.6% female veterans. Sample was primarily white 66%, 14% Hispanic and 14% black. The average age	A mixed-method study approach, quantitative data was collected at baseline, and longitudinally 3 and 6 months after the expressive writing interventions.<br>Participants were asked to complete four 20-minute writing sessions over 10 days after their baseline assessment.<br>Each piece of writing was qualitatively thematically coded.	Identity disruption was written about in the expressive writing activities. Four themes were found through thematic analysis:<br>1- Loss of purpose and meaning<br>2- Disconnection between past, present and future selves<br>3- Role disruption<br>4- Loss of self-worth<br>"For the purposes of subsequent quantitative analyses, identity disruption was coded with a "1" if identity disruption was present and a "0" if identity disruption was absent, regardless	Satisfaction with life- Measured at baseline, and 3, and 6 months with Diener's 5-item Satisfaction with life Scale, a common measure of subjective wellbeing.<br><br>Reintegration difficulty was also assessed with the Military to Civilian Questionnaire.	49% (n=121) of the participants had at least one of the four writing samples that was coded with a identity disruption theme.<br>27% of participants reported feeling a loss of meaning or purpose after returning from deployment. Disconnecting from the military left them feeling unfulfilled, questioning meaning of their work and lives.<br>27% indicated feelings of disconnect between their past, present and future selves, they expressed feeling cut off or estranged from their past selves, or alternatively trapped in the past an unable to move forward.<br>14% reported feelings of loss of self-worth, through expressing doubt about their past accomplishments.<br>Table 4 reports association between	Large sample size followed longitudinally enhances generalisability of findings and evidence trustworthiness. Rich data from qualitative expressive writing. Identity disruption was coded from the open-ended written qualitative responses, but there is no way to determine if some experienced identity disruption but did not write about it. The sample likely over emphasises negative experiences as the included sample had all self-reported that they experienced at least a little bit of difficulty, and the study did not include those who had no difficulty. The length of

Author(s) (Year). Title.	Study Aims/Research Question(s)	Study Population	Study Design and Method	Exposure variables	Outcome Variables	Main Study Findings	Study Strengths/Limitations & Future Directions
		was 38.8 years and average time since deployment was 6.2 years. Unclear how deployment and discharge intersect.<br><br>Country: United States of America		of which of the four [qualitative] theme(s)were reflected in their responses."		identity disruption and life satisfaction (r = -.15). Table 6 reports that "those who reported identity disruption tended to report lower life satisfaction at baseline." See also figure 3. Satisfaction with life remained stable over the follow-up period. Identity disruption and age were significantly associated, individuals reporting identity disruption were slightly younger than individuals not reporting identity disruption. Those who reported identity disruption tended to report lower life satisfaction. Veterans reporting identity disruption had lower levels of life satisfaction and more reintegration difficulty at baseline. Results indicated that many veterans in their 20's, 30's and 40s reported identity disruption themes. Returning to civilian life may pose a threat to temporal identity integration because it involves major disruptions across several of these domains that are salient in established adulthood and midlife.	time since deployments for the veterans may have impacted the results as the participants have had significant time to adjust to their reintegration and were more distant from it. Study is also unclear about how returning from deployment and military discharge are explicitly connected (terms were used interchangeably in places).<br><br>The study did not include many participants over the age of 60, so firm conclusions cannot be made about a later-in life sample. Future research should examine how the timing and developmental context of disruptive life events might buffer or exacerbate their effects on identity integration across the life span.
Park et al. (2021). Post-9/11 military veterans' adjustment to civilian life over time following separation from service	"We aimed to describe the positive as well as negative aspects of psychosocial well-being and to include a more diverse population than have many extant studies, including a large portion of female	n=402 veterans completed 3 survey assessments over a 12-month period. Participants were eligible if they were a veteran who served in Iraq, Afghanistan, or surrounding waters, and had separated from the military within the last 5	Quantitative Analysis of longitudinal data from the SERV study. (The SERV cohort study examined the post deployment experiences of post-9/11 veterans with an emphasis on examining possible gender differences).	Meaning in life measured with by the Meaning in Life Questionnaire – A 5 item presence subscale from the Meaning in life Questionnaire. Gender differences were examined.	Mental and Physical health-related quality of life- measured by the short-form health study (SF-12). The SF-12 yields two subscales: physical composite score (PCS) and mental composite score (MCS, on which	Transition appears to unfold over a period of many months as veterans experience a variety of challenges when returning to civilian life. There were no demographic differences (e.g., gender) on new mental and physical health problems across the study, or in baseline meaning-in-life. Veterans' levels of meaning in life were comparable to those of the civilian counterparts,	Large sample followed longitudinally enhanced evidence trustworthiness. However, the study uses a convenience sample and potentially not representative of the population of other veterans who have served. Many veterans were also lost to follow-up during the

Author(s) (Year). Title.	Study Aims/Research Question(s)	Study Population	Study Design and Method	Exposure variables	Outcome Variables	Main Study Findings	Study Strengths/Limitations & Future Directions
	veterans as well as veterans who did not engage in combat". Several research questions were examined, including 1- "How do veterans baseline quality of life and changes in quality of life over time differ among veterans based on demographic characteristics?"	years and was unlikely to be redeployed. Gender: reported as 60% men and 40% women, and mostly white (78%). Average age of 35.8 years old. Average time between military discharge and baseline interview was 40.8 months. Country: United States of America	The larger study comprised of 9 waves of data collection: at enrolment (baseline), 3, 6,9,12,18,24,30 and 36 months. Due to only a small amount remaining in the study after 12 months, data from participants who completed at least 3 waves of data by the 12-month follow-up were included for this current study.		higher scores mean better mental health QoL).	this could potentially be explained by the sense of meaning and purpose that serving in the military gives. Meaning, emotional and instrumental social support and mental quality of life were all positively associated at baseline (i.e., higher meaning in life was associated with greater mental QoL). By 12 months life meaning was slightly higher, but differences between 3-6 and 9 months fell short of significance, and when adjusting for covariance results remained similar. Mental health quality of life was slightly higher from 3 months onwards. Meaning in life may be a protective factor against stressors experienced when undergoing transition. "Meaning in life in the context of distress may be a protective factor in preventing worsening mental health among veterans." (p.2089)	longitudinal analysis and results may be vulnerable to attrition bias. Important components were also not measured such as life satisfaction which may have additional impacts on baseline scoring. Interview administration may have also introduced bias in the responses. The veterans in the study on average were already well into their transition and the time period tracked is a somewhat arbitrary point relatively early in longer-term adjustment. Demographic differences in life meaning are understudied and warrant further attention in future research. Future research should follow veteran from discharge through the first years of reintegration to assess social support trajectories.

Author(s) (Year). Title.	Study Aims/Research Question(s)	Study Population	Study Design and Method	Exposure variables	Outcome Variables	Main Study Findings	Study Strengths/Limitations & Future Directions
Reed (2001). Soldier, civilian or both? Identity continuity and discontinuity among ex-soldiers and implications for therapy.	"The current research sought to investigate the notion of threat to identity through disruption of the identity principles of continuity, decisiveness, self-esteem and efficacy of soldiers. It also sought to examine the ex-soldiers' attitudes to seeking therapeutic intervention to make recommendations for clinical practice and support".<br>This thesis contained multiple hypotheses, but the first was the most relevant to this review:<br>Fulfilment of identity continuity, decisiveness, self-esteem and self-efficacy will be linked to satisfaction with life.	n=121 ex-soldiers. Gender: all were male. Females were excluded from the study. Mean age of 46.37 years. 73.6% had served in trickle-post basis and 26.4% had served in a non-trickle-post basis. The mean length of military service was 18.6 years and the mean number of years since leaving the Army was 10.9 years.<br><br>Country: United Kingdom	Quantitative cross-sectional design, self-report survey. Convenience sample.	Centrality of the 'soldier' or 'civilian' identities- measured through 3 separate single-item scales.<br>Importance of the 'soldier 'identity- 1 item instrument measurement tool.<br>Importance of the 'civilian' identity- 1 item measurement tool.<br>Continuity of identity- 6-item measurement tool using 5-point Likert scale.<br>Rating of identity elements- 14 item scale (factor analysis generated distinctiveness, efficacy, and self-esteem scores related to both social interaction and individual recognition)<br>Length of service and desire to leave the army were also assessed as predictor variables.	Satisfaction with life scale- measured by Deiner et al, (1985) 5-item scale.	"In partial support of hypothesis 1, the analysis revealed that distinctiveness acquired as a soldier in both social and group interaction and individual recognition factors, self-esteem acquired as a soldier in the individual recognition factor were good predictors of satisfaction with life" (p.199). Identities indicative of soldier distinctiveness/social, soldier distinctiveness/individual and soldier self-esteem/individual were significant predictors of satisfaction with life. The findings indicated that as the length of years served increased, satisfaction with life increased. Those who wanted to leave the army at the time of discharge reported higher levels of satisfaction with life compared to those who had not wanted to leave the army.	Limitations: The study was limited to men, and did not consider female perspectives. The sample may have been non-representative due to the method of attracting participants.

Author(s) (Year). Title.	Study Aims/Research Question(s)	Study Population	Study Design and Method	Exposure variables	Outcome Variables	Main Study Findings	Study Strengths/Limitations & Future Directions
Stevenson (2020). Psychotherapy for veterans navigating the military-to-civilian transition: A case study	"The case presented in this paper will detail my attempts at addressing the range of issues associated with one veteran's military-to-civilian transition."	n=1 27-year-old veteran, male gender. Joined the marines at age 18 who served for two years. Country: United States of America	Qualitative Single Case study. Participant was referred through a psychologist colleague who was being treated specifically for depression.	Identity – based on case notes<br>Purpose – based on case notes	Wellbeing and overall functioning – based on case notes.	The military gave Jason a purposeful social identity that provided him with a needed sense of belonginess, direction and role responsibility. His transition out of the military represented a loss of prescribed social and vocational identity and left him facing a familiar challenge of creating a life of meaning and purpose within a narrowly defined social space. He had demonstrated significant challenges in functioning due to his sense of purposelessness, loneliness and negative core self-image. Therapy was approached through a relationship lens.<br>While also integrating a positive psychology/positive masculinity approach to psychotherapy for men. Jason was able to identify a valued career path of his choosing that corresponded with decent work that would satisfy his needs for social connection, self-determination which would lead to job satisfaction and overall wellbeing. He also began volunteering at his local school which in turn made him feel empowered to create a life he valued for himself and gave him a sense of satisfaction. Jason developed a new narrative for himself, recognising his limitation and adapting by expanding possibles roles and identities for himself that had previously been unacceptable (such as volunteering).<br><br>For veterans lacking purpose,	Limitations: This single case study focused on one male participant. The results may not be an accurate representation of the overall veteran population. The case study did not identify any specific measures of identity or wellbeing that may have been used. The patient was initially referred due to a diagnosis of depression rather than for wellbeing issues or loss of identity and purpose.<br><br>Future research would strengthen the results by expanding the number of participants and increasing the use of measurement tools to give more robust data.

Author(s) (Year). Title.	Study Aims/Research Question(s)	Study Population	Study Design and Method	Exposure variables	Outcome Variables	Main Study Findings	Study Strengths/Limitations & Future Directions
						meaning and social connectedness in the civilian world, the need for identity is paramount. It is imperative that clinical seek to help veterans develop a healthy identity that can adapt to the changing dynamics of life.	

Author(s) (Year). Title.	Study Aims/Research Question(s)	Study Population	Study Design and Method	Exposure variables	Outcome Variables	Main Study Findings	Study Strengths/Limitations & Future Directions
Wakefield et al. (2024). Brothers and sisters in arms: a mixed-methods investigation of the roles played by military support and social identity processes in the mental health of veterans during the transition to veterancy.	"This research aimed to use the Social Identity Model of Identity Change (SIMIC) to better understand the transition to veterancy"<br>Research Predictions: Veterans who feel they were highly supported by military during their period of transition will be more likely to (i) maintain connections with preexisting groups (social identity continuity) and (ii) join new groups (social identity gain), which will provide key psychological resources that support adjustment- notably of enhanced feelings of meaning in life, self-esteem and sense of personal control. Finally, we predict that greater perceived access to these psychological resources will be associated with better mental health (i.e. lower loneliness, depressive symptomology and	Study 1: n=210 Veteran participants. Gender: 162 males and 26 females, 2 'others'. Mean age of 50.38 years, range from 19-88 years old.<br>Study 2: subsample of n=14 Veteran participants from Study 1. Gender: 10 males, 4 females. Mean age of 57.29 years aged between 22-78 years.<br><br>Time since discharge not mentioned.<br><br>Country: United Kingdom	A mixed methods explanatory sequential design.<br>Study 1: Online survey to collect quantitative data (cross-sectional).<br>Study 2: Qualitative investigation. Semi-structured online interviews with 14 of the original study 1 participants to answer the following questions based off Study 1 results:<br>1- What are participants perceptions regarding the support they received from the military during their veterancy transition, and do they feel this encouraged them to join new groups?<br>2- What are the participants experiences of maintaining pre-transition group memberships into the veterancy transition, and do they feel that military support encouraged this maintenance?<br>3- What are the participants perceptions regarding	Study 1:<br>Meaning in life- measured with the 3-item meaning in life questionnaire short form. This was combined with questions on personal control and self-esteem to create a composite 'psychological resources' score.<br>Connection with maintained groups was measured with the four-item maintained group memberships sub-scale of the Exeter Identity Transition Scales.<br>Connection with new groups was measured with the four-item new group memberships sub-scale of the Exeter Identity Transition Scales.<br>Level of perceived social support during transition also self-reported.<br>Study 2:<br>Qualitative interview questions were related to social group memberships, psychological resources, and	Study 1:<br>Satisfaction with life was measured with Diener's et al 5-item scale.<br>Other outcomes measured were depression and loneliness.<br><br>Study 2: The findings from the interviews were presented as themes (in which wellbeing was represented)<br><br>Four themes:<br>1- Military support may encourage group gain, but not always<br>2- Military support may not encourage group maintenance<br>3- Group maintenance benefits veteran wellbeing<br>4- Group gain benefits veteran wellbeing<br>5- Group benefit veteran wellbeing by unlocking psychological resources	Study 1:<br>Maintaining group membership, new group membership, and having psychological resources (meaning, control and esteem) were all positively associated with greater satisfaction with life/wellbeing. In the mediation pathway models, maintained group membership indirectly predicted mental health/wellbeing via psychological resources. Furthermore, perceived military transition support indirectly predicted mental health/wellbeing serially via two mediators: new groups joined post-transition and psychological resources.<br>Extending the SIMIC research, we showed that both the continuity and maintenance pathways are in operation in the data, with participants who maintained group memberships and who joined new groups experiencing better mental health/wellbeing via psychological resources and enhanced meaning in life, self-esteem and personal control. Those who felt highly supported by the military tend to extend their social networks by joining new groups which in turn positively predicts health/wellbeing. Whilst retrospective perceived military transition support predicted new group memberships, it did not predict maintained group memberships. Study 1 highlighted the important role played by groups (new and maintained) in predicting veteran wellbeing.	Strengths: This research provides a novel and important insight into the veteran's transition through the lens of SIMIC. Mixed-methods enabled examination of associations identified in larger sample to be more deeply probed through qualitative interviews.<br><br>Limitations:<br>Study 1- convenience sample using a cross-sectional survey design, so causal relationships could not be explored. The support question was asked in general terms and did not ask participants to reflect on the extent to which they had received that support specifically in veterancy transition.<br>Future research should address this by more explicitly measuring transition related support.<br>As the participants had already transitioned this could have led to inaccuracies of the reflection of the support received. The transition timeframe was unclear.<br>Future research should include pre-transitioning service people to allow for real time data collection to

Author(s) (Year). Title.	Study Aims/Research Question(s)	Study Population	Study Design and Method	Exposure variables	Outcome Variables	Main Study Findings	Study Strengths/Limitations & Future Directions
	higher satisfaction with life (SWL)). i.e., the prediction that meaning in life will be associated with higher satisfaction with life was part of their hypotheses in relation to 'psychosocial resources'.<br>Study 2: To shed further light on and engage in further interpretation of study 1 findings. The study aimed to explore lived experienced of the transition as well as the factors that supported and/or hindered it.		whether maintaining groups and/or joining new groups affected their wellbeing?<br>4- Are group memberships (whether maintained or new) perceived by participants as providing important psychological resources?<br>Interview data was thematically analysed.	wellbeing during veterancy transition.		Study 2:<br>Provides qualitative support for the quantitative models presented in study 1. The data highlights the participants experiences of the wellbeing-related benefits they derived from group maintenance/gain as well as the unlocking of psychological resources such as meaning, esteem and control. Participants talked about lacking meaning after losing their military identity, and how group memberships boosted their sense of purpose, making them feel better and improving their wellbeing.<br>Overall conclusions:<br>SIMIC processes remain observable even in a context where pre-transition identity is likely to be chronically salient and life defining. The findings suggest that the norms of a strong military identity may be incompatible with the norms of civilian groups which can lead to difficulties obtaining wellbeing enhancing support from these groups.<br>Perceived/real transition support positively impacts group maintenance or joining new groups which unlocks psychological resources that positively impact SWL.	occur.

Author(s) (Year). Title.	Study Aims/Research Question(s)	Study Population	Study Design and Method	Exposure variables	Outcome Variables	Main Study Findings	Study Strengths/Limitations & Future Directions
Waldhauser et al. (2021). Purpose after service through sport: a social identity-informed program to support military veteran well-being.	"The overall purpose of this study was to evaluate the feasibility and acceptability of a social-identity informed group-based physical activity program that was designed to promote social connectivity, health and wellbeing among veterans" (p.425). The study examined military veterans experienced of Purpose After Service through Sport (PASS) which is a sport-based program in Canada, underpinned by the social identity approach.	n=12 participants who had attended the PASS program at least once, with a mean age of 39.83 years of age, with a mean of 15.63 years of service. Time since discharge not mentioned. All participants were reported as 'male' (sex vs gender unspecified), and Canadian however one was an ex British soldier. Majority served in the Army, n=10, one from the navy and one had served in both army and navy. Participants varied in their rank, which ranged from junior no commissioned members to junior officers.<br><br>Country: Canada	A qualitative study evaluating an intervention program, using semi structed interviews and reflexive thematic analysis.<br>Questions and probes focused on the participants military background and personal background, involvement and experiences of the program and program recommendations. Interviews lasted from 30-78 minutes. Inductive and deductive thematic analysis were used to identify patterns within the data.	Shared military identity was described by interview participants, along with peer support for social connection.	The findings from the interviews were presented as themes. Three themes with subthemes:<br>1- Benefits derived from participating: Healthy body, healthy mind, experiencing social connection and providing it for others, Connection to resources and/or employment opportunities<br>2- Shared Military Identity as a Means of Social Connection: Shared values, Morals, and Personal Characteristics (including shared sense of purpose), Normalisation of bonding over ball hockey and a beverage<br>3- Structural features of the PASS Program: Simple structure of the program, Different from other programs supporting veterans, positive wellbeing and environment. A fourth theme was related to program recommendations.	The study found that having a shared sense of identity supported a sense of social connectivity and had positive mental wellbeing impacts. Interactions among veterans reported to result in positive social benefits that extended beyond the program. Findings support the use of social identity-based peer support programs as a potentially useful means of supporting veteran wellbeing during the transition to civilian life. Although the PASS program was not overly structured to support mental health (covertly the support of veteran's wellbeing was targeted) a number of participants experienced positive mental health outcomes as a result of their participation which can be seen through the healthy body, healthy mind and shared military identity as a means of connection themes.	Strengths: An in-depth analysis of a novel social identity informed sport program. Inclusivity of the program, seeking to reach out to all veterans not just injured or ill.<br>Limitations: No women participation in the program. Only one sport offered, likely other sports would increase participation of veterans.<br>Future research should examine the efficacy/effectiveness of the PASS program to support effective transitions and quality of life outcomes among military veterans using causal (quantitative) research designs.

Supplementary Table S3

Definitions of Key Terms in the Included Studies

Study	Identity	Purpose	Wellbeing	Transition	Veteran
Barnett et al. (2022). Transitioning to civilian life: The importance of social group engagement and identity among Australian Defence Force veterans.	Personal identity refers to how an individual defines themselves (the 'I' and 'me') as unique and with individual differences compared with other in-group members'	Not defined	Not defined	Not defined	Not defined
Brewster, et al (2021). A point of connection? Wellbeing, the veteran identity of older adults.	Identity, as a concept, is defined here as both a self-perception (how an individual sees themselves within the world) and an outward presentation (how and individual defines themselves in relation to others).	Not defined	Our broad definition of wellbeing takes into account the relational nature of wellbeing, which 'emerges collectively, rooted in different domains in life as well as through our biographies'. Wellbeing is therefore rooted in relationships and interactions, as well as individual and so is affected both by a person's understanding of their life in their social context, and their access to social contract and perceived support.	Not defined	A veteran is defined as a person who served in the active military, naval, or air service, and who discharged or released under conditions other than dishonourable.
Burkhart & Hogan. (2015). Being a female veteran: A grounded theory of coping with transitions.	Not defined	Not defined	Not defined	Not defined	Not defined
Carra et al. (2023). Rural former service members participate in meaningful occupations to 'fill the void' after military service.	Not defined	The subjective sense made of and significance felt about ones' existence.	Not defined	Not defined	Not defined
Gnall et al. (2023). Life meaning and mental health in post-9/11 veterans: the	Not defined	Not defined	Not defined	Not defined	Not defined

Study	Identity	Purpose	Wellbeing	Transition	Veteran
mediating role of perceived stress.					
Flack & Kite. (2021). Transition from military to civilian: identity, social connectedness, and veteran wellbeing.	Identity was defined using the social identity theory and self-categorisation theory, stating that both theories explain the formation and dynamic nature of identity in a real or perceived social context.	Not defined	Not defined	Not defined	Not defined
Keeling (2024). Stories of transition: U.S. Veterans' narratives of transition to civilian life and the important role of identity.	Not defined	Not defined	Not defined	Not defined	Not defined
Lancaster et al. (2018). Validation of the Warrior Identity Scale in the Chicagoland Veterans Study	The degree to which soldiers and officers are motivated and willing to internalise the expressed values and goals of the armed forces	Not defined	Not defined	Not defined	Not defined
Lee et al. (2020). Correlates of perceived military to civilian transition challenges among Canadian Armed Forces veterans.	Not defined	Not defined	Not defined	Not defined	Not defined
Mitchell et al. (2020) Identity disruption and its association with mental health among veterans with reintegration difficulty.	Identity can be conceptualised broadly as the sense of self: the roles, traits, goals values, beliefs and experiences that add up to create and individuals unique place in the world.	Not defined	Not defined	Not defined	Not defined

Study	Identity	Purpose	Wellbeing	Transition	Veteran
Park et al. (2021). Post-9/11 military veterans' adjustment to civilian life over time following separation from service	Not defined	Not defined	Psychosocial wellbeing encompasses far more than just a lack of psychopathy (e.g. PTSD, anxiety, insomnia) and includes resources and indicators of positive health (social support, meaning in life, and health related quality of life).	Not defined	Not defined
Reed (2001). Soldier, civilian or both? Identity continuity and discontinuity among ex-soldiers and implications for therapy.	Not defined	Not defined	Not defined	Not defined	Not defined
Stevenson. (2020). Psychotherapy for veterans navigating the military-to-civilian transition: A case study	Not defined	Not defined	Not defined	Not defined	Not defined
Waldhauser et al. (2021). Purpose after service through sport: a social identity-informed program to support military veteran well-being.	Social identity- their sense of self as a member of a social group. They define who we are and provide us with belonging, meaning and a sense of purpose. Individuals derive a sense of meaning from the groups with which they identify.	Not defined	Not defined	Not defined	In Canada, veterans include any former members of the Armed forces who successfully underwent basic training and were honourably discharged.
Wakefield et al. (2024). Brothers and sisters in arms: a mixed-methods investigation of the roles played by military support and social identity processes in the mental health of veterans during the transition to veterancy.	Not defined	Not defined	Not defined	A move from one way of life (serviceperson) to another (veteran)	Not defined
